# From Microscale to Macroscale: Nine Orders of Magnitude for a Comprehensive Modeling of Hydrogels for Controlled Drug Delivery

**DOI:** 10.3390/gels5020028

**Published:** 2019-05-15

**Authors:** Tommaso Casalini, Giuseppe Perale

**Affiliations:** 1 Biomaterials Laboratory, Institute for Mechanical Engineering and Materials Technology, SUPSI—University of Applied Sciences and Arts of Southern Switzerland, Via Cantonale 2C, Galleria 2, 6928 Manno, Switzerland; giuseppe.perale@supsi.ch; 2 Institute for Chemical and Bioengineering, Department of Chemistry and Applied Biosciences, ETH Zurich, Vladimir-Prelog-Weg 1-5/10, 8093 Zurich, Switzerland; 3 Department of Surgical Sciences and Integrated Diagnostics, Orthopaedic Clinic-IRCCS Ospedale Policlinico San Martino, Faculty of Biomedical Sciences, University of Genova, Largo R. Benzi 10, 16132 Genova, Italy

**Keywords:** hydrogels, mathematical modeling, drug delivery, molecular dynamics, multiscale modeling

## Abstract

Because of their inherent biocompatibility and tailorable network design, hydrogels meet an increasing interest as biomaterials for the fabrication of controlled drug delivery devices. In this regard, mathematical modeling can highlight release mechanisms and governing phenomena, thus gaining a key role as complementary tool for experimental activity. Starting from the seminal contribution given by Flory–Rehner equation back in 1943 for the determination of matrix structural properties, over more than 70 years, hydrogel modeling has not only taken advantage of new theories and the increasing computational power, but also of the methods offered by computational chemistry, which provide details at the fundamental molecular level. Simulation techniques such as molecular dynamics act as a “computational microscope” and allow for obtaining a new and deeper understanding of the specific interactions between the solute and the polymer, opening new exciting possibilities for an in silico network design at the molecular scale. Moreover, system modeling constitutes an essential step within the “safety by design” paradigm that is becoming one of the new regulatory standard requirements also in the field-controlled release devices. This review aims at providing a summary of the most frequently used modeling approaches (molecular dynamics, coarse-grained models, Brownian dynamics, dissipative particle dynamics, Monte Carlo simulations, and mass conservation equations), which are here classified according to the characteristic length scale. The outcomes and the opportunities of each approach are compared and discussed with selected examples from literature.

## 1. Introduction

Hydrogels are hydrophilic cross-linked polymer matrices, able to absorb high amounts of water, up to several times of their dry weight. When the dry matrix is placed into the solvent, water starts diffusing through polymer chains, causing matrix swelling but not polymer dissolution, because of the cross-linked structure. A statistical description of their structure is usually performed by means of three parameters: Molecular weight between adjacent cross-links *Mc*, distance between two adjacent cross-links (also referred as mesh size) ξ, and cross-links density *ρ_c_*.

Starting from the seminal work of Wichterle and Lim in 1960 [1], interest and enthusiasm about hydrogels in biomedical field have never diminished and this is due to their peculiar combination of attractive properties. They are soft materials characterized by a large water content, which make them similar to living tissues and thus give them a potentially intrinsic biocompatibility. Hydrogel properties can be tailored by properly changing, e.g., polymer composition or cross-link density, leading to a wide range of applications from tissue engineering to devices for controlled drug release. In this regard, the focus of the present review relates to the fact that hydrogels can be loaded with active molecules, proteins, and genes, which can be protected from a potentially harsh environment at the release site, where they are delivered with a tunable release rate [2,3,4]. On top of that, hydrogels can be designed as smart materials that can respond to changes in the external environment; indeed, polymer matrices can be tailored in order to modify their behavior according to pH, temperature, shear stress, etc. [5]. 

Because of the impact of hydrogel bulk properties on its behavior, a sparkling modeling activity naturally arose with a twofold purpose. On the one side, modeling allows rationalizing and understanding the main phenomena behind the experimental behavior; on the other side, theoretical predictions are a powerful tool for simulating the effect of the main design parameters on system behavior in terms of, e.g., release rate over time. Model outcomes can contribute to optimize a time- and money-consuming experimental activity and support the development of new pharmaceutical formulations.

In this regard, the first milestone is constituted by the work of Paul J. Flory [6], who developed a comprehensive theoretical framework for the analysis of gels; for his contributions in the field, Flory won the Nobel Prize in chemistry in 1974. After about 70 years, hydrogels modeling has embraced not only the increased availability of computational resources, which makes accessible the numerical solution of detailed by complex models, but also methods like molecular dynamics, dissipative particle dynamics, or Monte Carlo simulations that provide a deeper detail at molecular level and offer a new understanding of the involved phenomena. Such methods can be seen as a “computational microscope” that allow a tailored design of the polymer matrix also at fundamental atomic scale. 

The purpose of this review is to guide the interested reader through the most employed modeling approaches, from full-atomistic simulations to mass conservation equations, from the molecular scale to the macro scale, highlighting the potentiality and the main outcomes of each method by discussing selected examples from literature.

## 2. Modeling Approaches—Brief Theoretical Background

In the following sections, the commonly employed approaches are classified according to the accessible length and time scales. On one side, there are molecular dynamics (MD) simulations, which provide a system description at the atomic level and whose characteristic length and time scales are nanometers and nanoseconds, respectively. On the other side, macroscale models exhibit the highest time and length scales (seconds and meters, respectively) but describe the system as a continuum, thus losing the molecular detail. In between, there are coarse-grained (or mesoscale) methods, which (at least partially) renounce the atomic detail but still keep a description at fundamental molecular level. Through the compromise of a simplified but physically consistent representation of the system under investigation, they aim at overcoming the limit of MD simulations by increasing the accessible time and length scales.

Because of the peculiar temporal and spatial resolution, each approach provides different insights concerning the system of interest, which synergistically contribute to a deeper understanding of the observed phenomena. A summary is provided in Table 1.

### 2.1. Molecular Dynamics Simulations

In molecular dynamics simulations, atoms are represented as mutually interacting hard spheres and electrons are not explicitly considered. Particle interactions are governed by a potential energy function, usually referred to as force field (FF). Molecular coordinates and velocities are obtained by integrating Newton’s equation of motion [7]:

(1) $$m_{i}\frac{d^{2}r_{i}}{dt^{2}}=F_{i}=-\nabla U\left(r\right)$$

where *m_i_* is the mass of the *i*-th atom, *r_i_* are the spatial coordinates of the *i*-th atom, *t* is time, *F_i_* is the force experienced by the *i*-th atom, and *U(r)* is the force field, which is an explicit function of all molecular coordinates *r*. 

Force fields usually take into account intramolecular bonded interactions (due to bond stretching, angle bending, and change in dihedral angles) as well as inter- and intra-molecular long-range interactions, that is, electrostatic and Van der Waals interactions. A 6/12 Lennard–Jones (LJ) potential is employed for Van der Waals interactions, while Coulomb law is usually adopted for electrostatic ones.

Force fields are extensively parameterized in order to best reproduce the outcomes from detailed quantum chemistry calculations at a high level of theory (structural properties, conformational energies) and/or experimental data [8]. There are different force fields available in literature, from general-purpose ones to FF specifically tailored for a given category of molecules, such as proteins, nucleic acids, lipid bilayers, and carbohydrates [8]. The choice of the right force field is fundamental, since the reliability of the results strongly depends on FF parameterization and accuracy.

MD simulations represent the ideal tool for those systems whose behavior is mainly due to non-covalent interactions. Environmental effects can be accounted for through the addition of explicit water molecules, ions, or other solute molecules. The effect of pH can be included by modifying the protonation states according to the acid dissociation constant value. 

Typical applications of MD simulations are the study of binding poses of small ligands bound to target proteins, drug permeation through lipid bilayers that mimic cell membranes, protein conformations, self-assembling of amphiphilic compounds, and interactions of nucleic acids with carriers [9,10,11,12,13,14]. 

The main outcomes of a simulation are molecular trajectories, whose subsequent post-processing allows obtaining structural information (number of hydrogen bonds, solvent and ions distribution around the solute, stacking of aromatic rings, etc.) as well as energetic information, such as interaction energies. 

Focusing on hydrogels, MD simulations are employed to characterize polymer conformation in water solution and the diffusion of small molecules in the matrix. The self-diffusion coefficient can be obtained from molecular trajectories by means of Einstein equation [15]:

(2) $$D=\underset{t\to \infty }{\lim}\frac{1}{2dt}{\left\langle [r\left(t\right)-r\left(t=0\right)\right]}^{2}\rangle$$

where *D* is the self-diffusion coefficient, *d* is the dimensionality of the system, *t* is time, and *r(t)* are the molecular coordinates of the solute at time *t*. The term inside angular brackets is the mean square displacement (MSD); that is, the squared distance traveled by the molecule at time *t*. Angular brackets indicate that MSD values are averaged over multiple solute molecules and/or multiple time origins. The limit indicates that Equation (2) is valid for time scales that are long enough so that Brownian motion regime is reached; that is, when MSD is a linear function of time and the self-diffusion coefficient can be obtained as a slope of the *t* versus MSD plot through linear regression. The attainment of Brownian motion regime can be checked through a log(*t*) versus log(MSD) plot; the slope obtained through linear regression must be very close or equal to one. 

In general, the mean square displacement as a function of time can be expressed through a power law:

(3) $$MSD\;\propto \;t^{\alpha }$$

Brownian regime corresponds to the value of exponent α equal to one. If α is lower than one, a subdiffusion regime takes place, while if α is higher than one, superdiffusion occurs. Experimental and computational works [16,17,18,19,20] pointed out that the diffusion of solutes in hydrogel can deviate from Brownian regime, leading to a sub- or superdiffusion regimen; in this case, Equation (2) is not valid. 

### 2.2. Coarse-Grained Models

The aim of coarse-grained (CG) models is to afford the simulation of complex systems by building a simplified representation that keeps the main peculiarities, such as the charge, the interplay between hydrophobic/hydrophilic effects, etc. A coarse-grained model allows for performing meaningful simulations for those systems that are intrinsically too complex for full-atomistic MD because of the limitations concerning time and length scales. This goal can be achieved by partially renouncing the atomic detail and enclosing groups of atoms in beads or interaction sites. This approach implies a loss of degrees of freedom that is counterbalanced by a reduced computational effort and the increased accessible time and length scales.

Coarse-graining procedure can be performed to different extents: Indeed, an interaction site can include a group of few atoms, an amino acid, a protein or a micro- or nanoparticle, according to the employed computational technique, and the phenomena under investigation. 

Coarse-grained simulations can be carried out by means of different approaches, characterized by the peculiar way with which molecular trajectories are computed and the forces experienced by the beads (*vide infra*). Beads interact with each other through a suitable potential energy function, which accounts for bonded and non-bonded interactions; while solvent can be either explicit (through the addition of solvent beads, which enclose a given number of solvent molecules) or implicit; in this case, the effect of the solvent is lumped in the parameterization of the potential energy function and/or included in additional terms, such as a friction force. 

The parameters involved in coarse-grained models are usually tuned in order to best reproduce the outcomes of a more detailed MD simulation and/or experimental data. Parameterization is often tailored for specific systems; this limits parameters transferability and implies that a new parameter set must be obtained for each system. In this regard, MARTINI force field [21,22] attracted a lot of interest for its straightforward parameterization procedure. MARTINI beads enclose a group of three or four heavy atoms and are classified in four categories: Charged, polar, apolar, and non-polar; additional sub-groups account for the different hydrogen bond capability or polarity. While parameters for bonded interactions must be determined through MD, a set of parameters for non-bonded interactions (expressed through a 6/12 Lennard–Jones potential) is already available for each bead, obtained in order to best reproduce thermodynamic properties like partition free energies. Such parameters can be further refined to improve the agreement with detailed MD simulations. 

Molecular trajectories are here computed by integrating Newton’s equation of motion and using MARTINI force field without additional terms. Solvent and ions can be explicitly included (a MARTINI water beads contain four water molecules). A set of parameters for simulations with implicit solvent, called Dry MARTINI, is currently available and validated only for lipid membranes [23].

Other popular methods for CG models are Brownian dynamics (BD) simulations, dissipative particle dynamics (DPD) simulations, or Monte Carlo (MC) simulations.

Brownian dynamics simulations are based on Langevin equation, which includes three contributions acting on the *i*-th particle: A systematic force *F^s^_i_* (that depends on particles coordinates), a frictional force *F^f^_i_* (that depends on particle velocity), and a random force *F^r^_i_* (that depends on time). Systematic forces are conservative and account for the mutual interactions between particles, while frictional forces are non-conservative and take into account the effect of the drag due to the solvent. The random force has a Gaussian probability distribution; it is responsible for the Brownian motion and acts as a white noise (that is, its time average is zero). In particular, *F^r^_i_* satisfies the following conditions:

(4) $$\langle F_{i}^{r}\left(t\right)\rangle =0$$

(5) $$\langle F_{i}^{r}\left(t\right)F_{j}^{r}\left(t^{\prime }\right)\rangle =2k_{B}T\zeta \delta_{ij}\delta \left(t-t^{\prime }\right)\delta$$

where *ζ* is the drag coefficient, *δ_ij_* is Kroenecker delta, *δ(t − t’)* is Dirac delta function, and *δ* is the unit second order tensor. Brownian dynamics represent the limit case of overdamped Langevin dynamics, where inertial term is neglected:

(6) $$m_{i}\frac{d^{2}r_{i}}{dt^{2}}=F_{i}^{s}\left(r\right)+F_{i}^{f}\left(v\right)+F_{i}^{r}\left(t\right)=0$$

(7) $$F_{i}^{s}\left(r\right)=-\nabla U\left(r\right)$$

(8) $$F_{i}^{f}\left(v\right)=-\zeta \frac{dr_{i}}{dt}.$$

In dissipative particle dynamics [24,25], particle trajectories are still computed from the Newton equation of motion; each *i*-th particle experiences a force that is a sum of three pair-additive components: A conservative force *F^c^_ij_*, a dissipative force *F^d^_ij_*, and a random force *F^r^_ij_*:

(9) $$m_{i}\frac{d^{2}r_{i}}{dt^{2}}=f_{i}=\sum_{j\neq i}F_{ij}^{c}+F_{ij}^{d}+F_{ij}^{r}.$$

Conservative force derives from the particles’ interaction potential (an elastic force for bonded interactions, a soft repulsion force for non-bonded interactions), dissipative force tries to damp the relative motion between particles, while random force is directed along the line connecting particles center. Dissipative and random forces are momentum conserving, and represent a minimal model to account for viscous forces and thermal noise between particles. Contrary to Brownian dynamics, each particle experiences a stochastic force that depends also on the other particles.

Monte Carlo (MC) simulations [7] aim at generating a representative ensemble of system configurations according to the chosen thermodynamic conditions. Particles still interact with each other according to a suitable potential (that accounts for both bonded and non-bonded interactions) but dynamics are not propagated through the Newton equation of motion and usually the Metropolis algorithm is employed, which can be summarized as follows:

- At simulation step *n*, the computed system energy is *U_n_*;
- At stimulation step *n + 1*, randomly-chosen particles attempt to perform a random displacement Δ*r*;
- A new energy value *U^n+1^*, deriving from such displacement, is computed;
- Displacement attempt is accepted with a probability:
  (10) $$acc\left(n\to n+1\right)=\min\left(1,\exp\left[-\frac{1}{k_{B}T}\left(U^{n+1}-U^{n}\right)\right]\right)$$
- In order to decide whether to accept or reject the random displacement, a random number *x* is generated from a uniform distribution in the interval [0, 1]. If *acc(n → n+1)* *≥ x*, the move is accepted; otherwise, it is rejected.

MC simulations cannot be employed to track time evolution of a system, but they can generate a physically consistent ensemble of configurations from which thermodynamic properties of interest, such as free energy, can be obtained. 

### 2.3. Macroscale Models

Macroscale models are based on fundamental mass, energy, and momentum conservation principles. In the drug delivery field, they are usually employed to highlight the main phenomena behind the release rate of an active molecule from a drug-loaded hydrogel and its quantitative estimation; polymer degradation can be also taken into account. For this reason, only mass balances are normally written; the system can be reasonably considered as isothermal (energy balance is not needed), while momentum conservation equation is needed only in particular cases, e.g., when the interest is focused on the calculations of stresses and deformations. 

Available mechanistic models can be classified according to the rate-determining step behind release mechanism. Although several phenomena contribute to the observed release rate, it is challenging to consider all of them in a mathematical model. On the one side, including only rate-determining phenomena reduces model complexity, achieving a good compromise between simplicity and a reasonable system description. On the other side, this allows reducing the number of parameters that must be estimated from experimental data. The inclusion of several factors and/or complex theories may lead to the determination of many parameters (that can also be strictly system-dependent) and thus to overfitting; that is, a good agreement between model results and experimental data due to the large set of estimated parameters and not necessarily to the consistency of the constitutive laws. 

When molecular diffusion is the rate-determining phenomenon, Fick’s first law can be conveniently used as a starting point for highly-swollen hydrogels:

(11) J=−D∇C

where *J* is the diffusive flux, *D* is the diffusion coefficient, and *C* is the concentration of the diffusant. In the simplest case, the diffusion coefficient is constant in time and space and the mass balance for the diffusant can be written as follows (Fick’s second law):

(12) $$\frac{\partial C}{\partial t}=D\nabla^{2}C=D\left(\frac{\partial^{2}C}{\partial x^{2}}+\frac{\partial^{2}C}{\partial y^{2}}+\frac{\partial^{2}C}{\partial z^{2}}\right).$$

The Laplacian operator can be written in Cartesian (as in Equation (12)), cylindrical, or spherical coordinates according to system geometry. For the sake of simplicity, one spatial coordinate is often considered; that is, where the gradients are more relevant (the radius for a sphere, the thickness for a slab, etc.). Mass balances can be analytically or numerically solved with suitable initial and boundary conditions. A uniform drug distribution is usually employed as initial condition, while the most common boundary conditions are the symmetry of concentration profile at the device center and a given concentration at device surface, or the continuity of mass fluxes if interphase mass transport resistance is accounted for. Analytical solutions are available in the literature for simple geometries (slabs, cylinders, spheres), constant diffusion coefficients, and sink conditions (drug concentration in the release medium equal to zero). 

In a more realistic situation, a diffusion coefficient can be a complex function of hydrogel structural parameters as well as diffusant, polymer, and water concentration, thus reflecting the complexity of the system. Time- and spatial-dependent diffusivity and/or complex geometries lead to the necessity of numerical solutions, although, nowadays, reliable results can be efficiently obtained with a standard personal computer. 

For diffusion-controlled systems, the challenge lies in a suitable expression for the diffusion coefficient. If the mesh size is much larger than the size of the diffusant, diffusivity values can be conveniently expressed using suitable values of porosity and tortuosity. On the other hand, when mesh and diffusant sizes are comparable, the drag due to the polymer chains must be accounted for. As reviewed [26], the modeling approaches can be mainly classified as follows:

- Models based on obstruction effects: Polymer chains are assumed motionless if compared with the diffusant, by virtue of their lower self-diffusion coefficient. Polymer matrix is thus modeled as a rigid and impenetrable network, whose effect is the increasing of the mean diffusive path. These models are suitable for small molecules and low polymer concentrations, while they do not provide reliable results at high polymer concentration, since solute/network interactions cannot be neglected anymore.
- Models based on hydrodynamic theories: Polymer chains are still assumed to be motionless with respect to the solute, which is modeled as a hard sphere moving at constant velocity in a continuum and experiencing a frictional drag, according to Stokes–Einstein formalism. Polymer chains enhance the frictional drag by slowing down the surrounding fluid.
- Models based on free volume theory: Free volume can be defined as the volume of the system at a given temperature minus the volume of the same system at 0 K or, in a more straightforward way, as the volume not occupied by matter. These models are based on the assumption that diffusion phenomena are governed by free volume arrangements that create pores and path where solute and solvent molecules can move.

Literature offers additional and more specific modeling strategies. Fatin-Rouge and coworkers developed and validated a model for the diffusion of ions in agarose gels, accounting for the effects of electrostatic interactions and the impact of changes in pH and ionic strength [27]. In addition, it should be taken into account that, if the initial drug concentration is above its solubilization limit, dissolved and undissolved drug coexist until drug excess is present into the system. In this case, drug solubilization dynamics could also play a role. 

**Figure 1:**
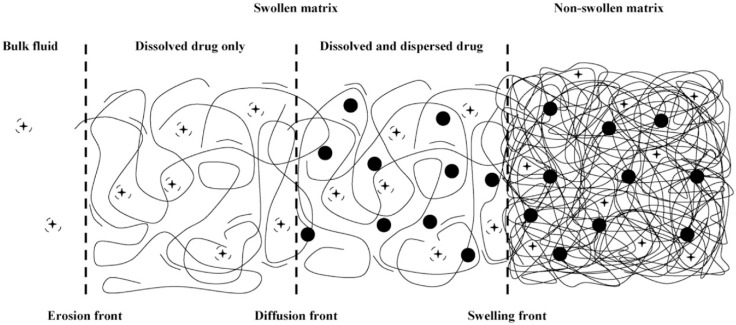
Representation of a swelling-controlled system with different zones separated by moving boundaries. Reprinted from Siepmann and coworkers [28], Copyright 2008 Elsevier.

Polymer swelling can also constitute a rate-determining step for drug release. In this case, the active compound is initially immobilized in a dry polymer matrix in a glassy state. When the device is put in contact with water or the release medium, solvent diffusion into polymer matrix leads to swelling and volume increase. As soon as a given system-specific solvent concentration is reached, chain relaxation or glass-to-rubbery phase transition takes place; chain mobility steeply increases, also enhancing drug mobility and thus promoting its release. Swelling-controlled devices can be modeled by dividing the system in different zones separated by moving fronts, as shown in Figure 1. On the right side, there is the inner core of the device (still in glassy state), while on the left side, there is the solvent (or the release medium). Starting from the left side of Figure 1, the solvent diffuses into the polymer matrix, and swelling as well as chain relaxation begin to occur. The boundary where these phenomena take place is the swelling front and discriminates the swollen portion of the matrix from the non-swollen one. The swelling front is not stationary but move inwards in time because of the solvent diffusion towards the core of the device.

In the swollen portion of the matrix, solubilized (black crosses) and insolubilized (filled black dots) drugs can coexist depending on the initial concentration of the active compound. It is possible to identify another boundary, the diffusion front, which separates the portion of swollen matrix that contains only dissolved drug molecules where both dissolved and undissolved molecules are present. If water-soluble matrices are used, a third boundary, the erosion front, can be identified. 

The controlling mechanism behind drug release can be assessed by comparing the characteristic time scale for diffusion τ_diff_ and the characteristic time scale for polymer relaxation τ_rel_ by means of a dimensionless group referred as Deborah number De:

(13) $$De=\frac{\tau_{rel}}{\tau_{diff}}=\frac{D\tau_{rel}}{\delta {\left(t\right)}^{2}}$$

where *δ(t)* is the time dependent thickness of the swollen polymer. Low De values (De << 1) are representative of diffusion-controlled systems (molecular diffusion is slower than chain relaxation) while high De values (De >> 1) indicate that the behavior of the system is governed by the swelling (molecular diffusion is faster than chain relaxation).

In chemically controlled systems, drug release rate can be related to one of the following aspects [29]:

- The drug is covalently linked to polymer chains through cleavable spacers, and the rate-determining step is the kinetics of bond cleavage;
- Drug release is mainly due to surface erosion;
- Drug diffusion is hindered by polymer chains and the rate-determining step is polymer degradation, which creates new and wider diffusive paths;
- The active compound is not covalently bound to polymer chains and the rate-determining step is the binding equilibrium.

In this framework, the modeling approach should couple drug diffusion with a suitable description of spacer cleavage kinetics, polymer degradation kinetics, or the affinity with the polymer matrix. 

## 3. Applications

The following sections aim at illustrating the strength of each discussed modeling approach. Because of the involved time and length scales, each method unavoidably provides different kinds of information. The atomic detail of molecular dynamics simulations allows for investigating transport phenomena (in terms of solute self-diffusion coefficient) and the environmental effects on chains conformation. Coarse-grained models are usually employed for evaluating the influence of some tunable degrees of freedom (polymer charge, ionic strength, ions charge, temperature, etc.,) on hydrogel structure, while macroscale models are useful for obtaining structural parameters and the release rate of a loaded active molecule. 

### 3.1. Molecular Dynamics Simulations

As mentioned, molecular dynamics simulations are mainly employed for the investigation of the impact of environmental conditions (water content, temperature, ionic strength, etc.,) and material formulation (chain composition, cross-link density, etc.,) on transport phenomena and chain conformation. Post-processing of molecular trajectories allows assessing the impact of solute/matrix interactions, the diffusion regime and, if the Einstein equation is valid, computing a self-diffusion coefficient that intrinsically contains all system peculiarities. The atomic resolution gives a detailed overview of system conformation and interactions with the solvent, in terms of, e.g., molecules distribution around polymer chains and the evolution of hydrogen bonds. 

Despite the detailed outcomes from MD simulations, their use is still limited to small mesh size values (around 10 nm) because of the requested computational effort and the intrinsic accessible length scale. Bigger systems can be conveniently investigated by means of coarse-grained models (*vide infra*). 

At full atomistic level, there are two main approaches adopted in the literature for the development of hydrogels molecular models. One method identifies a basic building block composed by three (or more) chain cross-linked each other, which are repeated in space by means of periodic boundary conditions; this allows for obtaining an infinite network that constitutes a reasonable statistical description of the matrix. The molecular model is usually assumed defect-free; that is, without dangling chains or self-loops. 

Chiessi et al. [30,31] developed a model of cross-linked polyvinyl alcohol (PVA) hydrogels, in order to study polymer solvation and the dynamics of water molecules inside the matrix. Radial distribution functions allowed identifying three domains: First solvation shell, second solvation shell, and bulk water. Solvent diffusion coefficient was computed at different temperature values (291, 303, 313, and 323 K) in each domain. 

Jang et al. [32] simulated the mechanical and transport properties of single network (SN) hydrogels made of polyethylene oxide (PEO) and polyacrylic acid (PAA) and a double network (DN) hydrogel with both PEO and PAA; water content is the same for all investigated systems (76% in weight terms). Stress/strain curves were obtained through uniaxial extension simulations, while transport properties were assessed by computing a self-diffusion coefficient for ascorbic acid and d-glucose. 

**Figure 2:**
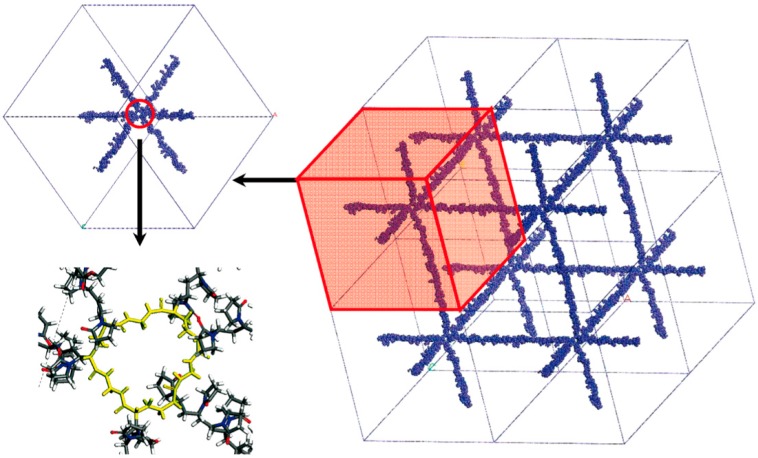
Schematization of the modeling approach adopted by Lee and coworkers. A building block that contains some cross-linked chains is repeated in space by means of periodic boundary conditions, obtaining a regular network. Adapted with permission from Lee et al. [33], Copyright 2009 American Chemical Society.

Lee et al. [16,33,34] focused their attention on hydrogels made of poly(*N*-vinyl-2-pyrrolidone-*co*-2-hydroxyethyl methacrylate) (PVP-*co*-HEMA) copolymer cross-linked with *N*,*N*’-methylene bisacrylamide (MBA); VP:HEMA ratio is equal to 37:13 for all systems. The modeling approach is depicted in Figure 2.

They found that stress/strain curves depend on water addition and the sequence of monomers in polymer chain; such behavior is due to the different rearrangement of HEMA monomers. If water content increases (from 20% to 80%), system behavior becomes less dependent on copolymer sequence. The self-diffusion of d-glucose and ascorbic acid also strongly depended on copolymer composition and water content at low hydration, showing anomalous subdiffusion at low water amounts (about 20%).

Jaramillo-Botero et al. [35] studied SN and DN hydrogels made of polyacrylamide (PAAM) and poly(2-acrylamido-2-methylpropanesulfonic acid) (PAMPS) cross-linked with MBA for cartilage scaffolding and analyzed the mechanical properties at different water content.

He and coworkers [36] found three water-dependent structural transitions of zwitterionic carboxybetaine methacrylate (CBMA) hydrogels. The authors subsequently studied the mechanical properties [37] of CBMA and OH-*p*-CBMA hydrogels, where additional hydrogel groups act as physical cross-linkers. Stress/strain curves showed that OH-*p*-CBMA systems have a greater elastic modulus than CBMA ones, because of the additional contribution given by hydroxyl groups with hydrogen bonding.

Pascal et al. [38] combined MD simulations and two-phase thermodynamics (2PT) method to characterize the thermodynamics of water molecules inside CBMA hydrogels at different hydration. 

Wu et al. [39] investigated the effect of cross-link density on the diffusion of rhodamine, water, and chloride ions in hydrogels made of polyethylene glycol diacrylate (PEGDA). A normalized diffusion coefficient was computed for water and solutes as a function of cross-linked density and compared with the outcomes of Amsden model (*vide infra*); predictions are in good agreement each other. 

Sun et al. [40] studied the pH-dependent interactions between a PVA hydrogel functionalized with short peptides (Glu-Asp-Pro-Trp, EDPW) and the monoclonal antibody trastuzumab (commercial name: Herceptin) used for the treatment of breast cancer. Two different pH values were considered (3 and 7) and the protonation states of the ligands and the antibody were modified accordingly. The performed simulations provided interesting insights but they are too short (12 ns) if compared to the time scale needed to observe relevant conformational changes in proteins.

In general, such simulations are very useful to study transport phenomena at the fundamental molecular level, complementing the existing theories. Anyway, their extensive use for network design is still hindered by a systematic experimental validation.

A second strategy for hydrogels simulation at the molecular level is placing one or more polymers chains (which can be cross-linked each other) in a simulation box filled with the solvent of choice. This technique is often employed to simulate the chain conformation of hydrogels based on poly(*N*-isopropylacrylamide) (PNIPAM), widely studied because of its thermoresponsive behavior. PNIPAM is characterized by a lower critical solution temperature (LCST): Below this temperature value, polymer/solvent interactions dominate, and chains are in coil conformation. Above LCST, polymer/polymer interactions become more relevant and chains assume a globular arrangement in the solvent. LCST value for PNIPAM is about 32 °C, close to physiological temperature, making this material a good candidate for biomedical applications [5].

Walter et al. [41] studied the conformation of a PNIPAM chain (composed by 30 monomer units) in pure water at different temperature, in order to reproduce the thermoresponsive behavior by checking the time evolution of chain gyration radius. Walter et al. also investigated conformational changes in mixtures of water and methanol with different composition and at different temperatures [42]. The employed computational protocol allowed reproducing the experimentally observed co-nonsolvency (i.e., PNIPAM chains have a coil conformation in pure water and methanol but a globular arrangement in a water/methanol mixture). 

Deshmukh et al. [43] studied the structure of PNIPAM hydrogels chemically cross-linked with MBA in water, at temperature values above and below the experimental LCST (300, 305, and 310 K) and with different cross-link densities. They found that chain collapse is less relevant for high cross-link density values, because of the additional constraint of the polymer structure. Deshmukh and coworkers subsequently studied the effect of the compound used for covalent cross-linking [44], considering *N*,*N*’-methylene bisacrylamide and ethylene glycol dimethacrylate (EGD). 

Alaghemandi and Spohr [45] also investigated PNIPAM chain collapse as a function of temperature, which ranges from 280 to 330 K, characterizing structural transitions.

Tönsing and Oldiges [46] simulated PNIPAM hydrogels cross-linked with MBA, focusing their analysis on the structure of polymer solvation shells; computed water self-diffusion coefficients at different temperatures are in good agreement with experimental data. 

Oliveira et al. [47] used MD simulations to compare the thermoresponsive behavior of PNIPAM and Poly(*N*-*n*-propylacrylamide) (PNnPAM), which exhibits a lower LCST (24 °C for PNnPAM, 32 °C for PNIPAM) and a more discontinuous transition with a steeper size change. The authors successfully reproduced the different LCST, which were explained.

Recently, Garcìa et al. [48] pointed out that standard MD simulation might not be able to provide an adequate sampling of PNIPAM chain conformation in solution, because of the high number of involved degrees of freedom and the intrinsic time scale limitations. Indeed, in order to collect suitable statistics from a simulation, coil-to-globule transition should be observed several times, and this can require very long simulations, up to microsecond time scale. From an experimental point of view, coil-to-globule transition occurs in the millisecond time scale, while the analogous information for the short oligomers used in standard MD simulations is not available. The system investigated by these authors is composed by two PNIPAM oligomers composed of 15 monomer units. Structural transition from the dissolved to the aggregated state is investigated by means of umbrella sampling (an enhanced sampling method), where a harmonic bias potential is added considering the distance of the centers of mass of the oligomers in the *xy* plane *d* as collective variable (i.e., the most relevant degree of freedom). The main outcome is the potential of mean force (PMF) *G(d)* as a function of the chosen collective variable, which shows the free energy difference between the dissolved and the aggregated states Δ*G_d→a_*.

**Figure 3:**
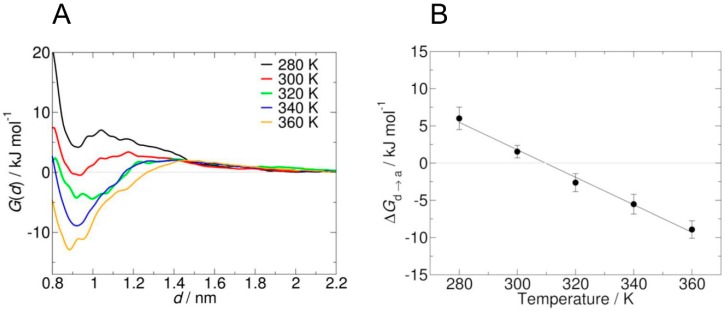
Potential of mean force of the transition between dissolved and aggregated state of poly(*N*-isopropylacrylamide (PNIPAM) in water at different temperatures, scaling mixing rules by a factor equal to 1.1 (**A**). Free energy change between dissolved and aggregate state at different temperatures (**B**). Reprinted from Garcìa et al. [48], Copyright 2018 Elsevier.

Notably, adopting OPLS force field with SPCE water models (like other authors) umbrella sampling reveals that water is a bad solvent for all investigated temperatures (from 280 to 360 K). The scaling of the mixing rule with a factor equal to 1.1 led not only to the reproduction of the structural transition, but also to a LCST value (309 ± 9 K) in good agreement with experimental data. PMF at different temperatures and the related Δ*G_d→a_* values are shown in Figure 3; a value of *d* equal to 0.9 nm indicates aggregate state, while at 2.2 nm polymer chains are not interacting anymore (solvated state). 

Du and Qian [49] studied the transition of a poly(*N*-isopropylacrylamide-*co*-ethylene glycol methacrylate) (PNIPAM-*co*-PEGMA) block copolymer in 1 M NaCl solution, highlighting the role of the interactions between the polymer and the salt. Chiessi et al. [50] investigated the thermoresponsivity of covalently cross-linked hydrogels made of poly(vinyl alcohol)/poly(methacrylate-*co*-*N*-isopropylacrylamide) (PVA-MA-NIPAM) for drug delivery purposes, with different network topologies. 

Focusing on other systems, Oldiges and Tönsing [51,52] analyzed water dynamics in polyacrylamide (PAAM) hydrogels cross-linked with MBA and swollen with a dilute aqueous acetonitrile (AAN) solution. The authors computed an environmentally dependent diffusion coefficient for water molecules; the ensemble-averaged values are in good agreement with experimental data. 

Jiang et al. [53] analyzed water structuring in cross-linked PVA matrices, while Zhang and coworkers [54] studied the structure of PVA gels swollen with water/ethanol mixtures with different compositions as well as solvent diffusion within the matrix. 

Paradossi and coworkers [55] investigated the chain mobility of chemically cross-linked polyvinyl alcohol/polymethacrylate (PVAMA) gels: They found that a temperature increase enhances network mobility with a composition-dependent behavior. Hydrophilic PVA chains exhibited a relevant mobility increase with temperature, while rigid PMA segments were essentially unaffected. 

Avila-Salas et al. [56] used MD simulations to study the pH-dependent behavior of a gel made of PVA, chemically-cross linked with maleic acid (MA), and functionalized with γ–cyclodextrin molecules (γ–CD). This is system is aimed at providing a controlled release of nifedipine (a calcium channel blocker) included into γ–CD. MD simulations were employed to study the influence of pH on gel structure; it was found that a low pH the matrix has a compact structure because of the protonation state of MA moieties, while at neutral pH, the repulsion between dissociated carboxyl groups creates an open gel structure, which enhances swelling and the release of nifedipine loaded into γ–CD.

Valdés, Avila-Salas, and coworkers [57,58] studied the adsorption of dimethoate and methamidophos (organophosphorus pesticides) from water solutions by means PVA hydrogels cross-linked with MA. Simulations at molecular level allowed highlighting the most important degrees of freedom for an optimal matrix design. 

Ou et al. [59] adopted MD simulations to study water adsorption at solvent/gel interface, focusing on matrices made of polyethylene glycol diglycidyl eether (PEGDGE) and polyoxyalkyleneamines as curing agent. Volumetric swelling was computed at different temperatures and degrees of cross-link. 

Baker et al. [60] proposed a computational protocol based on molecular dynamics simulations in implicit solvent to describe proteins translocation through nanoporous hydrogels suspended in microfluidic devices. The computed free energy change related to translocation was in good agreement with experimental observation.

Simulations at molecular level proved to reproduce the main peculiarities of the investigated systems. Again, a systematic experimental validation seems to be the missing piece of the puzzle that would allow an optimized design of the matrix at molecular scale. 

As recently reviewed [9], molecular dynamics attracted a lot of interest for the investigation of supramolecular polymers, which can also lead to the formation of supramolecular gels. In this regard, MD simulations act as a computational microscope that provides an atomic detail on the specific non-covalent interactions (hydrogel bonds, π–π stacking, hydrophobic effects, etc.,) that result in the formation of supramolecular assemblies as well as the impact of chemical modifications and environmental conditions (pH, ionic strength, etc.). 

Atomistic simulations are usually employed to elucidate the main phenomena behind the early stages of gelation, which involve the initial formation of supramolecular aggregates, such as fibers. The simulation of the gelation process and the consequent formation of a disordered entangled polymer network require time and length scales not accessible by molecular dynamics simulations; in addition, it is challenging to account for concentration-dependent effects. 

Two main approaches can be identified: Top-down and bottom-up. In top-down simulations, a given number of monomers is initially assembled in a reasonable guess configuration, which is subsequently equilibrated through MD simulations (or coarse-grained model). This approach is employed if a consistent starting arrangement can be formulated from experimental insights and/or high-level simulations. On the other hand, bottom-up simulations are performed by randomly placing a given number of monomers in a simulation box (along with solvent molecules and ions, if needed) and allowing the spontaneous self-assembling. This approach is feasible with simple monomers, i.e., with a low structural complexity; bottom-up simulations of complex systems are challenging or even unfeasible and a top-down approach becomes a forced choice.

In general terms, it should be taken into account that long simulations are required for the attainment of reasonably equilibrated structures, which might be long-lived metastable states and not representative of a minimum energy conformation. In this regard, the use of enhanced sampling methods can alleviate this issue and increase the robustness of the model outcomes. In addition, top-down and bottom-up approaches are not limited to MD simulations but are also applied to CG models. 

Angelerou et al. [61] studied, from both an experimental and a computational point of view, the self-assembling of a cytosine-based gelator. Fiber structures suggest that small hydrophobic drugs may be encapsulated in the core, thus opening new opportunities for drug delivery applications. 

Eckes and coworkers [62] developed a fluorenylmethoxycarbonyl (Fmoc)-conjugated alanine-lactic acid (Ala-Lac) compound, and studied its self-assembling in water. Sathaye et al. [63] focused their attention on MAX1 β-hairpin peptide, which forms supramolecular networks in water environment through fibril entanglement and fibril branching. The authors designed and proposed a new peptide based on MAX1, referred to as LNK1, where they substituted nonturn valine with 2-naphthylanaline and alanine, which exhibit larger and smaller side chain steric hindrance, respectively. The aim is to obtain a specific “lock and key” complementary arrangement in the hydrophobic core and reduce fibril branching. Molecular simulations showed that LNK1 fibrils are more rigid and more resistant to structural defects than MAX1 ones because of the induced specific packing in the hydrophobic core. Model outcomes were confirmed by experimental data. An additional comprehensive analysis at atomic scale of MAX1 fibril structure is presented by Miller et al. [64].

### 3.2. Coarse-Grained Models

In this framework, the term “coarse-grained models” (CG) is employed for those approaches where there is a loss of degrees of freedom with respect to a full atomistic representation of the system. Groups of atoms are enclosed in beads or interaction sites, which interact each other through a potential energy function and can experience additional effects, such as drag forces or random forces, according to the chosen simulation method.

Coarse-grained models represent a further approximation of the system, but this is an unavoidable price to pay for affordable and meaningful simulations of complex systems and/or to overcome the limitation imposed by the time and length scales of full atomistic models. CG simulations are often carried out with implicit solvent (whose effects are included in the parameterization of beads interactions), further simplifying system description but drastically decreasing the computational effort. Hydrogels are usually modeled as perfect and defect-free networks, highlighting a building block that is repeated in space by means of periodic boundary conditions as already discussed for molecular dynamics simulations (*vide supra*). Nanogels are represented as small networks, which can change their conformation according to the environmental effects (salt concentration, ion valence, etc.).

The obtained results are often compared with the analogous predictions of available analytic theories, in order to assess their validity and the impact of the underlying assumptions; in some cases, improved analytic models are also proposed according to simulations outcomes. 

In general, despite the loss of the atomic resolution and the implicit solvent representations, CG models are able to adequately reproduce, in a trend-wise manner, the effects of important degrees of freedom involved in network design at a reasonable computational cost. CG simulations can suggest some guidelines for system optimization, in terms of, e.g., chain length or number of hydrophobic/hydrophilic groups that must be included, concretely supporting device development.

In the following sections, relevant examples from literature are discussed according to the adopted method.

### 3.3. Coarse-Grained Molecular Dynamics

In CG MD simulations, beads interactions are determined only by the potential energy function, i.e., the force field. MARTINI force field attracted a lot of interest for its straightforward parameterization procedure (*vide supra*) and it is employed for the simulation of polymer matrices as well, as discussed in the following examples. 

Gautieri et al. [65] simulated the diffusion of benzene in PVA matrices; non-bonded parameters were optimized through full atomistic MD simulations. The computed benzene self-diffusion coefficient was in very good agreement with experimental data. Although the specific system under investigation is not of interest in the biomedical field, the proposed methodology can be extended to solvents and solutes commonly employed for drug delivery. 

Zadok and Srebnik [66] adopted MARTINI to investigate hydrogels made of protein-imprinted polymers, using acrylic functional monomers and lysozyme and cytochrome as templates. Their work was focused on protein dynamics and the changes to the gel deriving from protein diffusion.

Salahshoor and Rahbar [67] determined swelling, water diffusion, and stress/strain curves for hydrogels made of PEGDGE cross-linked with polyoxyalkyleneamines at different water contents. 

Zhang et al. [68] studied the self-assembling of Fmoc-d-Ala-d-Ala dipeptide, which forms hydrogels in water environment. Simulations showed that π–π stacking is the main driving force that leads to the formation of supramolecular nanofibers. Brown et al. [69] combined MD and CG MD for the study of carboxybenzyl-protected diphenylalanine (zFF), which forms rigid and self-healing hydrogels in solvent combinations with high water content.

Frederix et al. [70] systematically analyzed the self-assembly propensity in water solution of all possible 8000 tripeptide combinations. After a first screening, the most promising candidates were selected according to their aggregation propensity (AP) and hydrophilicity-corrected AP (AP_H_). The formation of nanostructures was studied in extended simulations for the chosen sequences; computational results were compared experimental data taken from literature. In addition, the authors synthesized and tested some of the promising peptides suggested by the in silico screening, finding a good agreement with model results. 

Focusing on other approaches, Fu et al. [71,72,73] employed an ePRIME coarse-grained force field for proteins in order to study extensively the self-assembling of peptide amphiphiles as a function of temperature and the extent of electrostatic interactions and hydrophobic effects. The authors obtained phase diagrams elucidating the obtained geometries for different combinations of the investigated conditions. 

Despite the loss of the atomic resolutions, CG simulations allowed elucidating the main driving forces behind aggregation, providing useful guidelines for network design.

### 3.4. Brownian Dynamics and Langevin Dynamics

Langevin dynamics and Brownian dynamics (also referred as overdamped Langevin dynamics, where the inertial term is neglected) are employed not only for the study of polymer conformation, but also for characterizing transport phenomena. A bead-and-spring model is usually adopted for polymer chains, representing each monomer as a single bead connected with the adjacent units with massless springs; bonded interactions are accounted for through a simple harmonic potential, or as an alternative, through the finite extension non-linear elastic (FENE) potential, widely employed in polymer simulations. Electrostatic interactions can be taken into account with Coulomb law, while the repulsion due to excluded volume effects is commonly included through Weeks–Chandler–Andersen potential. Hydrophobic effects can be accounted for as well, through an appropriate LJ-like attractive potential. 

Kosovan et al. [74] used Langevin dynamics in order to study the swelling of polyelectrolyte gels as a function of salt concentration, chain length, and degree of ionization. They compared the results with the Katchalsky and Michaeli theory (which explicitly includes electrostatic effects) for the partitioning of salt between gel and bulk solution, finding a good agreement across the chosen range on investigated parameters.

Mann et al. [75,76] extended their previous study of polyelectrolyte gels in good solvents to swelling equilibrium in a bad solvent. The authors provided a structure diagram, where different conformations are depicted as a function of fraction of charged monomers and Bjerrum length, which in this framework, identifies the strength of electrostatic interactions. 

Ghelichi and Qazvini [77] used Langevin dynamics to study the formation of a supramolecular hydrogel, whose monomers are hydrophilic charged blocks capped with hydrophobic moieties, by varying the charge of the midblock, counterion valence, the stiffness, and the length of hydrophobic groups.

Several studies have been devoted to the characterization of transport phenomena and the mechanisms that must be included for a physically consistent description. Hansing and Netz [78] developed a theoretical framework to include hydrodynamic effects in BD simulations; the model was applied to study the diffusion of a nanoparticle in a cubic gel.

Sandrin et al. [79] focused their attention on the diffusion of dextran nanoparticles in polyacrylamide hydrogels, coupling an experimental investigation with BD simulations.

Pei and coworkers [80] expressed the diffusion coefficient of a particle in a gel as the product of two terms, namely *D_EM_*, which accounts for hydrodynamic interactions and *S*, which includes short range steric interactions. The authors adopted BD simulations to compute the second term for rods and wormlike chains that mimic DNA.

Kvarnström et al. [81] studied dendrimers diffusion in hydrogels; simulations showed that obstructions effect alone could not reproduce experimental data, which could be better described by accounting for solute/matrix interactions. 

Zhou and Chen [82] simulated tracer diffusion in hydrogels by varying network porosity, chain stiffness, cross-link density, and electrostatic interactions, but neglecting hydrodynamic interactions. 

Tabatabaei et al. [17] analyzed in detail the mechanisms behind the attainment of anomalous diffusion regimes by simulating tracer diffusion in weakly cross-linked hydrogels, highlighting the deviations from the expected Brownian motion regime. 

Rapp et al. [83] synthesized a physically cross-linked hydrogel made of engineered triblock protein composed of two identical coiled-coil domains connected through a water soluble midblock; a network is formed through the association of the coiled-coil segments. The authors developed a theoretical framework where a protein can be present in three states, assumed to be at equilibrium: Free (i.e., not bound but freely diffusing in the water-filled pores), dangling (only one coiled-coil domain is associated to the network), and bound (both coiled-coil segments are involved in the network formation). BD simulations were coupled with experimental activity to study chain mobility and compute the two equilibrium constants. Model outcomes confirmed the experimental observations. 

### 3.5. Dissipative Particle Dynamics

Dissipative particle dynamics simulations emerged as a method of choice, because they allow for modeling complex fluids in a simple way by imposing suitable interactions between the particles and for higher time scales than the ones offered by molecular dynamics. Polymer chains are usually represented through a bead-and-spring model (*vide supra*), while bonded interactions are accounted for by a harmonic potential. Notably, DPD simulations can be also carried out with explicit solvent beads. 

Chen and Yong [84] developed an osmotic ensemble method for DPD aimed at studying hydrogel swelling in aqueous environment, imposing a constant solvent chemical potential. Simulation results are in agreement with the well-established Flory–Rehner theory (*vide infra*).

Nikolov and coworkers [85] examined the swelling and deswelling kinetics of a neutral microgel, as well as structural transitions. They found that, during swelling, the microgel maintains a homogeneous structure, while deswelling causes network coarsening and the formation of chain bundles. 

**Figure 4:**
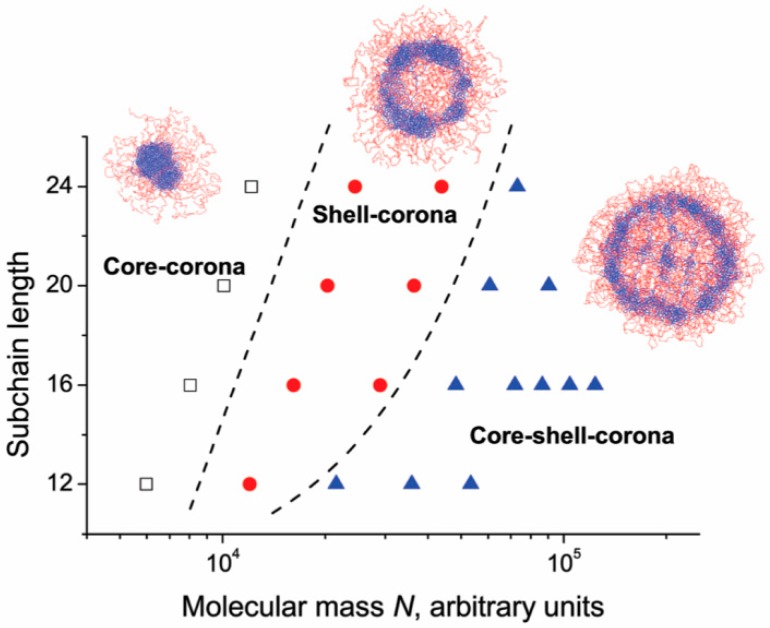
Interpenetrating polymer networks (IPN) microgel structure as a function of molecular mass and subchain length. Adapted from Rudyak and coworkers [86], Copyright 2018 Royal Society of Chemistry.

Rudyak et al. [86] studied the structure of microgels made of an interpenetrating polymer network constituted by a collapsed and a swollen subnetwork. Simulation showed how microgel structure changes by varying the number of subchains (260–2300) and the number of monomers for each subchain (12–24). The authors identified three possible arrangements: Core–corona, shell–corona, and core–shell–corona, as shown in Figure 4.

Masoud and Alexeev [87] used DPD to simulate hollow microgel capsules, which are able to swell or deswell in a stimuli-dependent manner, and the release of loaded linear macromolecules and nanoparticles. The authors investigated how the release rate changes for swelling and deswelling capsules.

Yong et al. [88] studied the formation of multilayered gels, where each layer is chemically cross-linked with the adjacent ones upon the addition of initiators, monomers, and cross-linkers. Notably, the authors employed a recently developed DPD-based method that is able to simulate a living copolymerization reaction scheme.

### 3.6. Monte Carlo Simulations

Monte Carlo simulations do not allow for tracking the time evolution of the system of interest, but it is possible to generate an ensemble of configurations consistent with the thermodynamic conditions. Coupled with appropriate bonded and non-bonded interaction potentials and usually performed with an implicit representation of the solvent, MC simulations are widely employed to study swelling equilibrium and network structure in different conditions. Polymer chains are represented with bead-and-spring coupled with a harmonic potential or FENE potential; non-bonded interactions are usually accounted for through Coulomb law (for electrostatic interactions) and WCA potential for short range repulsion due to excluded volume effects. 

Edgecombe and Linse [89] compared the behavior of single network and interpenetrating network hydrogels, as a function of chain length and network charge. They studied the swelling equilibrium and computed stress/strain curves applying uniaxial extension along z direction.

Ahualli et al. [90] examined the interactions between neutral nanogels with different values of chain length, employing different interaction potentials.

Quesada-Pérez et al. [91] compared the size-exclusion partitioning of neutral solutes in hydrogels obtained through MC simulations with the outcomes of Ogston’s model and pore model, commonly employed for their simplicity. Pérez-Mas and coworkers [92] subsequently extended the analysis by including also the contribution of hydrophobic adhesion and its interplay with excluded volume effects. The obtained results can suggest some guidelines for network design in order to maximize the loading of hydrophobic active compounds.

MC simulations attracted a lot of interest for the investigation of polyelectrolyte hydrogels [93,94,95,96]. In a series of works, Edgecombe and Linse [97,98,99] systematically examined the effects of salt, oppositely charged macroions, polymer polydispersity, and network defects on swelling equilibrium.

Quesada-Pérez and coworkers focused their attention on thermoresponsive charged nanogels [100,101,102,103,104]. First, they examined the structures of the network by changing temperature and counterions valence. Model results highlighted that charged nanogels can form hollow structures, where charged monomers are exposed on the inner and the outer surfaces. Then, the authors studied the effect of counterions valence and nanogel charge on the swelling of the thermoresponsive network. Simulations suggest that the valence of counterions strongly influences the structure of the nanogel, since trivalent counterions cause a shift of the transition temperature to lower values. The authors subsequently employed MC simulations to derive the forces between charged nanogels, taking into account charge distribution, chains flexibility, network topology, and the nonexistence of a perfect spherical surface.

### 3.7. Macroscale Models

Macroscale models are currently the method of choice for an optimal device design, since they allow determining the most important design parameters (in terms of geometry, drug loading, etc.,) and their impact on the release profile. After a systematic and careful validation, such models can be rationally employed to understand how the desired release rate can be obtained through the available degrees of freedom, optimizing experimental activity in terms of time and costs.

The starting point for macroscale models is Flory–Rehner theory [6], which allows evaluating structural properties of hydrogels without ionic moieties. Flory–Rehner theory states that, when an amorphous dry matrix is placed in a solvent, it is subjected only to two forces: A thermodynamic force related to polymer/solvent mixing and an elastic force that hinders chain stretching during swelling. The system is at equilibrium when these forces balance each other; in terms of Gibbs free energies [2,3,5]:

(14) $$\Delta G_{total}=\Delta G_{mixing}+\Delta G_{elastic}$$

where Δ*G_total_* is system free energy, Δ*G_mixing_* accounts for the spontaneous polymer/solvent mixing, and Δ*G_elastic_* is the contribution of the elastic force. Δ*G_mixing_* also includes the affinity between the matrix and the surrounding molecules, which is usually expressed by means of the polymer/solvent interaction parameter *χ_1_*. Equation (14) can be rewritten in terms of chemical potentials through differentiation with respect to the number of solvent molecules at constant temperature and pressure:

(15) $$\mu_{1}-\mu_{1,0}=\Delta \mu_{mixing}+\Delta \mu_{elastic}$$

where *μ_1_* and *μ_1,0_* are the chemical potential of the solvent inside the gel and in the surrounding, respectively. The change in chemical potential due to the mixing Δ*μ_mixing_* can be expressed using heat and entropy of mixing, while the change due to elastic forces Δ*μ_elastic_* can be obtained from rubber elasticity theory. At equilibrium, the difference between chemical potentials is zero; equating Δ*μ_mixing_* and Δ*μ_elastic_*, *M_c_* can be expressed as follows:

(16) $$\frac{1}{M_{c}}=\frac{2}{M_{n}}-\frac{\left(\frac{\upsilon }{V_{1}}\right)\left[\ln\left(1-\upsilon_{2,s}\right)+\upsilon_{2,s}+\chi_{1}\upsilon_{2,s}^{2}\right]}{\left(\upsilon_{2,s}^{\frac{1}{3}}-\frac{\upsilon_{2,s}}{2}\right)}$$

where *M_n_* is the molecular weight of the polymer before cross-linking, υ is the specific volume of the poymer in the amorphous state, *V_1_* is the molar volume of the solvent, and *υ_2,s_* is the polymer volume fraction in the swollen state. Equation (16) is valid when an already cross-linked dry polymer matrix is immersed in a solvent; if cross-linking process is performed when the polymer is already in solution, Equation (16) has been modified as follows:

(17) $$\frac{1}{M_{c}}=\frac{2}{M_{n}}-\frac{\left(\frac{\upsilon }{V_{1}}\right)\left[\ln\left(1-\upsilon_{2,s}\right)+\upsilon_{2,s}+\chi_{1}\upsilon_{2,s}^{2}\right]}{\upsilon_{2,r}\left[{\left(\frac{\upsilon_{2,s}}{\upsilon_{2,r}}\right)}^{1/3}-\frac{1}{2}\frac{\upsilon_{2,s}}{\upsilon_{2,r}}\right]}$$

where *υ_2,r_* is the polymer volume fraction in the relaxed state, i.e., after cross-linking but before swelling. The presence of the solvent modifies the change in chemical potential related to the elastic force. 

If ionic moieties are present in the polymer network, there is an additional contribution Δ*G_ionic_* to the change in Gibbs free energy:

(18) $$\Delta G_{total}=\Delta G_{mixing}+\Delta G_{elastic}+\Delta G_{ionic}.$$

Differentiation of Equation (18) with respect to the number of solvent molecules at constant temperature and pressure leads to:

(19) $$\mu_{1}-\mu_{1,0}=\Delta \mu_{mixing}+\Delta \mu_{elastic}+\Delta \mu_{ionic}.$$

The change in chemical potential Δ*μ_ionic_* due to the ionic moieties strongly depends on the ionic strength and the type of ions present in the system. Peppas and coworkers have developed suitable expressions that account for the presence of charged groups for both anionic and cationic hydrogels [105]:

(20) $$\begin{array}{l} \frac{V_{1}}{4I}\left(\frac{\upsilon_{2,s}^{2}}{\upsilon^{2}M_{r}^{2}}\right){\left(\frac{K_{a}}{10^{-pH}+K_{a}}\right)}^{2}\\ \;\;\;\;=\left[\ln\left(1-\upsilon_{2,s}\right)+\upsilon_{2,s}+\chi_{1}\upsilon_{2,s}^{2}\right]+\left(\frac{V_{1}}{\upsilon M_{c}}\right)\left(1-\frac{2M_{c}}{M_{n}}\right)\upsilon_{2,r}\left[{\left(\frac{\upsilon_{2,s}}{\upsilon_{2,r}}\right)}^{1/3}-\left(\frac{\upsilon_{2,s}}{2\upsilon_{2,r}}\right)\right] \end{array}$$

(21) $$\begin{array}{l} \frac{V_{1}}{4I}\left(\frac{\upsilon_{2,s}^{2}}{\upsilon^{2}M_{r}^{2}}\right){\left(\frac{K_{b}}{10^{pH-14}-K_{a}}\right)}^{2}\\ \;\;\;\;=[\ln\left(1-\upsilon_{2,s}\right)+\upsilon_{2,s}+\chi_{1}\upsilon_{2,s}^{2}]\\ \;\;\;\;+\left(\frac{V_{1}}{\upsilon M_{c}}\right)\left(1-\frac{2M_{c}}{M_{n}}\right)\upsilon_{2,r}\left[{\left(\frac{\upsilon_{2,s}}{\upsilon_{2,r}}\right)}^{1/3}-\left(\frac{\upsilon_{2,s}}{2\upsilon_{2,r}}\right)\right] \end{array}$$

where *I* is ionic strength, *M_r_* is the molecular weight of the repeating units, and *K_a_* and *K_b_* are acid and base dissociation constants, respectively. Hydrogel porosity is determined by the mesh size ξ, defined as the average distance between two adjacent cross-links:

(22) $$\xi =\alpha {\left({\bar{r}}_{0}^{2}\right)}^{1/2}$$

where α is the elongation ratio of polymer chains and *(r^2^_0_)^1/2^* is the root mean square unperturbed end-to-end distance of polymer chains between two adjacent cross-links. If swelling is isotropous, the elongation ratio can be related to the volume fraction of swollen polymer:

(23) $$\alpha =\upsilon_{2,s}^{-1/3}.$$

The unperturbed end-to-end distance can be obtained through the Flory characteristic ratio *C_n_* (whose values are tabulated for many polymers of interest) and bond length along polymer backbone *L*:

(24) $${\left({\bar{r}}_{0}^{2}\right)}^{1/2}=L{\left(C_{n}N\right)}^{1/2}$$

(25) $$N=2\frac{M_{c}}{M_{r}}$$

where *N* is the number of links per chain. Combining Equations (22)–(25) leads to:

(26) $$\xi =\upsilon_{2,s}^{-1/3}{\left(\frac{2C_{n}M_{c}}{M_{r}}\right)}^{1/2}L.$$

In order to obtain a reliable estimation of the mesh size, *υ_2,s_* must be experimentally determined in the solvent of choice, taking into account the influence of environmental conditions such as ionic strength and pH. *M_c_* can be subsequently evaluated with the most suitable expression (Equations (16), (17), (20) and (21)) and eventually the mesh size can be obtained through Equation (26). An analogous theoretical framework has been developed also for interpenetrating polymer networks (IPN), based on three main assumptions: Homogeneous behavior, IPN interactions, and independent network behavior [5]. A theoretical description for a thermoresponsive network is also discussed in the literature [5].

Focusing on drug release, the modeling approaches can be classified according to the rate-determining phenomenon, as explained in the previous sections (*vide supra*). Water-swollen hydrogels are usually in a rubbery state due to the decrease of glass transition temperature. If the system is reasonably homogeneous (i.e., structural discontinuities and non-swollen glassy regions are absent or negligible), diffusion can be described as Fickian, with a suitable expression for diffusivities that accounts for matrix structure, drug size, water content, polymer composition, and environmental conditions. 

As discussed, the rate-determining phenomenon (diffusion or swelling) can be firstly discriminated through the values of Deborah number. Peppas and coworkers [106] developed a simple empirical power law that allows determining the principal release mechanism starting from release data:

(27) $$\frac{M_{t}}{M_{\infty }}=kt^{n}$$

where *M_t_* and *M_∞_* are the cumulative amount of drug released at time *t* and infinite time, respectively, *k* is a proportionality constant and *n* is an exponent that discriminates system behavior; notably, Equation (27) is valid only up to 60% of released drug. If the device is a thin film, an exponent equal to 0.5 indicates that Fickian diffusion takes place, while if *n* is equal to one, the release is controlled by swelling. Intermediate values indicate the superimposition of swelling and diffusion (also referred as anomalous transport). The values of exponent *n* for different geometries are available in the literature and reported in Table 2 [107].

Another semi-empirical model has been discussed by Peppas and Sahlin [108]:

(28) $$\frac{M_{t}}{M_{\infty }}=k_{1}t^{m}+k_{2}t^{2m}$$

where *k_1_* and *k_2_* are proportionality constants related to Fickian diffusion and Case II transport contributions, respectively, and *m* is a constant. 

### 3.8. Diffusion-Controlled Systems

If diffusion is the rate-determining process, drug release can be conveniently described through a mechanistic model, i.e., solving Equation (12) with proper initial and boundary conditions and a suitable expression for the diffusion coefficient. It is usually assumed that the drug is uniformly dispersed in the hydrogel before release onset, while boundary conditions specify the values of concentration or fluxes at the interface or in the bulk. Commonly adopted boundary conditions are symmetry of the concentration profile at the center of the device and continuity of mass fluxes or a given drug concentration at the hydrogel/release medium interface. If the diffusion coefficient is constant in time and space, device volume is constant in time and for simple geometries, Equation (12) can be analytically solved. Fu and coworkers [109], for example, proposed an analytical solution for cylindrical geometries that accounts for mass transport along radial and axial coordinates:

(29) $$\frac{M_{t}}{M_{\infty }}=1-\frac{8}{h^{2}r^{2}}\overset{\infty }{\underset{m=1}{\sum }}\alpha_{m}^{-2}\exp\left(-D\alpha_{m}^{2}t\right)\times \overset{\infty }{\underset{n=1}{\sum }}\beta_{n}^{-2}\exp\left(-D\beta_{n}^{2}t\right)$$

(30) $$J_{0}\left(r\alpha \right)=0$$

(31) $$\beta_{n}=\frac{\left(2n+1\right)}{2h}\pi$$

where *h* is the half-length of the cylinder, *r* is the radius, *J_0_* is zero-order Bessel function, and *m* and *n* are integers. There are several attempts in literature to obtain more realistic diffusivity values that are correlated with hydrogel structure, solute size, etc. [26,110,111]. Amsden [111] has discussed the most relevant models as function of the underlying theory (*vide supra*), which are here summarized following his treatise. Focusing on free volume theory, Peppas and Reinhart [112] proposed the following expression:

(32) $$\frac{D_{g}}{D_{0}}=k_{1}\left(\frac{M_{c}-M_{c}^{*}}{M_{n}-M_{c}^{*}}\right)\exp\left(-\frac{k_{2}r_{s}^{2}}{Q-1}\right)$$

where *D_g_* is the diffusion coefficient in the hydrogel, *D_0_* is the diffusion coefficient in pure solvent at infinite dilution, M_c_^*^ is a critical molecular weight between cross-links, below which a drug molecule with size *r_s_* cannot diffuse through the matrix, *k_1_* and *k_2_* are constants related to polymer structure, and *Q* is the degree of swelling. Lustig and Peppas [113] developed an expression that explicitly includes the mesh size ξ:

(33) $$\frac{D_{g}}{D_{0}}=\left(1-\frac{r_{s}}{\xi }\right)\exp\left[-Y\left(\frac{\upsilon_{2,s}}{1-\upsilon_{2,s}}\right)\right]$$

(34) $$Y=\frac{\gamma \pi \lambda r_{s}^{2}}{v_{f,w}}$$

where *Y* is the ratio between the critical volume required for a successful translational movement of the solute and the average free volume per molecules of the liquid, *γ* is a numerical factor used to correct for overlap of free volume available to more than one molecule, *λ* is the jump length, and *v_f,w_* is the free volume per water molecule. The sieving factor (1 − *r_s_/ξ*) reflects the fact that only solute molecules whose size is smaller than mesh size can effectively diffuse through the matrix. 

Hydrodynamic theories use the Stokes–Einstein equation as a starting point for computing solute diffusivity. In this framework, the solute is modeled as a hard sphere, whose size is much larger than solvent one, which moves at constant velocity in a continuum and experiences a frictional drag. Polymer chains are assumed to be motionless and to increase the frictional drag of the solute by slowing down the surrounding fluid; therefore, the efforts are devoted to a suitable description of the frictional effect. Cukier [114] proposed the following formalism for hydrogels with rigid chains:

(35) $$\frac{D_{g}}{D_{0}}=\exp\left[-\left(\frac{3\pi L_{c}N_{A}}{M_{n}\ln\left(\frac{L_{c}}{2r_{f}}\right)}\right)r_{s}\upsilon_{2,s}^{1/2}\right]$$

where *L_c_* is polymer chain length, *N_A_* is Avogadro number, and *r_f_* is the radius of polymer fiber; for homogeneous hydrogels (that is, with high chain mobility), Cukier proposed the following expression:

(36) $$\frac{D_{g}}{D_{0}}=\exp\left(-k_{c}r_{s}\upsilon_{2,s}^{0.75}\right)$$

where *k_c_* is a constant for a given polymer/solvent system. Phillips and coworkers [115] employed Brinkman equation for a flow through porous media in order to compute the frictional coefficient, assuming no-slip conditions at solute surface and constant fluid velocity far from solute; the polymer is modeled as randomly-oriented rigid fibers:

(37) $$\frac{D_{g}}{D_{0}}={\left[1+{\left(\frac{r_{s}^{2}}{k}\right)}^{1/2}+\frac{1}{3}\frac{r_{s}^{2}}{k}\right]}^{-1}$$

(38) $$k=0.31r_{f}^{2}\upsilon_{2,s}^{-1.17}$$

where *k* is the hydraulic permeability; the here-employed correlation has been derived by Jackson and James [116]. In obstruction theories, polymer chains form a rigid impenetrable network, which increases the mean diffusive path. Ogston et al. [117] developed the following formula:

(39) $$\frac{D_{g}}{D_{0}}=\exp\left[-\frac{r_{s}+r_{f}}{r_{f}}\upsilon_{2,s}^{1/2}\right].$$

The authors assumed that solute diffusion can be rationalized as a succession of unit steps in random directions, which do not take place if a solute molecule encounter the polymer network, modeled as straight long fibers with a negligible width. The unit step is set equal to the root mean square average diameter of spherical spaces residing between fiber networks.

Johansson and coworkers [118] modeled the polymer network through a given number of cylindrical cells; each cell contains an infinite polymer rod centered in a cylinder of solvent with a given radius. Fick’s first law is solved in the cell in order to obtain an average diffusivity, while the global one referred to as the entire gel is calculated by summing up the number of cells with a given radius multiplied by the average diffusivity in that cell. Cell radii distribution is computed through an expression for the distribution of spherical spaces in a random network of straight fibers. The final expression is given by:

(40) $$\frac{D_{g}}{D_{0}}=e^{-\alpha }+\alpha^{2}e^{\alpha }E_{1}\left(2\alpha \right)$$

(41) $$\alpha =\upsilon_{2,s}{\left(\frac{r_{s}+r_{f}}{r_{f}}\right)}^{2}$$

where *E_1_* is the exponential integral. Another expression was developed in order to best reproduce the outcomes from Brownian dynamics simulations:

(42) $$\frac{D_{g}}{D_{0}}=\exp\left[-0.84\alpha^{1.09}\right].$$

The model of Tsai and Strieder [119] has been adapted in order to provide the best agreement with Brownian motion simulations:

(43) $$\frac{D_{g}}{D_{0}}={\left(1+\frac{2}{3}\alpha \right)}^{-1}$$

where α is given by Equation (41). Amsden [120] developed a model where solute diffusion is assumed to be a stochastic process; the diffusant is able to move through the matrix according to the possibility to find a succession of openings through the network, which are large enough to accommodate the molecule, whose size is determined by tis hydrodynamic radius. Solute diffusivity is given by:

(44) $$\frac{D_{g}}{D_{0}}=\exp\left[-\pi {\left(\frac{r_{s}+r_{f}}{k_{s}\upsilon_{2,s}^{1/2}+r_{f}}\right)}^{2}\right]$$

where *k_s_* is a constant for a given polymer/solvent system that accounts for chain flexibility. Successful approaches were also obtained by combining hydrodynamic theory and obstruction effects. Johnson and coworkers [121] merged the obstruction description of Johansson et al. [118] with Equation (14), obtaining:

(45) $$\frac{D_{g}}{D_{0}}=\frac{\exp\left[-0.84\alpha^{1.09}\right]}{\left[1+{\left(\frac{r_{s}^{2}}{k}\right)}^{1/2}+\frac{1}{3}\frac{r_{s}^{2}}{k}\right]}.$$

Clague and Philips [122] adopted the model of Tsai and Strieder for the obstruction effect, while they computed the hydrodynamic contribution by means of numerical simulations based on slender-body theory:

(46) $$\frac{D_{g}}{D_{0}}={\left(1+\frac{2}{3}\alpha \right)}^{-1}\exp\left[-\pi \upsilon_{2,s}^{0.174ln\left(59.6\frac{r_{f}}{r_{s}}\right)}\right].$$

Because of the underlying assumptions, the model of Clague and Philips can be used only for hydrogels with stiff polymer chains, such as agarose gels. A summary is provided in Table 3.

### 3.9. Swelling-Controlled Systems

If swelling is the rate-determining phenomenon, the complexity of the formulaically description increases because of the presence of the moving boundary (which separates the swollen and the non-swollen portion of the matrix) and the volume increase over time. A typical example in the literature is constituted by devices made of hydroxypropyl methylcellulose (HPMC). 

Peppas et al. [123] developed a model for drug diffusion in swellable matrices; in particular, the system under investigation is a cylindrical device placed in an inert support and immersed in a swelling medium, where a diffusant is released from the upper base surface. Swelling is assumed to be one-dimensional and to occur along cylinder axis. The starting point is constituted by the mass balances for the drug in the non-swollen matrix *C_1_*, the drug in the swollen portion of the matrix *C_2_*, and the swelling agent *S*:

(47) $$\frac{\partial C_{i}}{\partial t}=D_{i}\frac{\partial^{2}C_{i}}{\partial x^{2}}\;\;\;\;\;\;\;\;\;i\;=1,2$$

(48) $$\frac{\partial S}{\partial t}=D_{s}\frac{\partial^{2}S}{\partial x^{2}}$$

where *D_1_*, *D_2_*, and *D_s_* are the diffusion coefficients of the drug in the non-swollen portion of the matrix, the drug in the swollen matrix, and the swelling agent, respectively, and *x* is a spatial coordinate. It is assumed here that the solute is initially uniformly dispersed in the matrix; boundary conditions can be written as follows at the matrix/inert support interface (*x* = 0), the time-dependent swelling front (*x* = x*), and the time-dependent polymer/swelling agent interface (*x* = *L*):

(49) $$\frac{\partial C_{1}\left(0,t\right)}{\partial x}=0$$

(50) $$C_{1}\left(x^{*},t\right)=C_{2}\left(x^{*},t\right)=C^{*}$$

(51) $$D_{1}\frac{\partial C_{1}\left(x^{*},t\right)}{\partial x}=D_{2}\frac{\partial C_{2}\left(x^{*},t\right)}{\partial x}$$

(52) $$\frac{\partial S\left(x^{*},t\right)}{\partial x}=0$$

(53) $$S\left(L,t\right)=S_{0}$$

(54) $$C_{2}\left(L,t\right)=C_{0}.$$

Equations (50) and (51) impose the continuity at the swelling front, Equation (52) states that the swelling agent diffuses only up to the swollen/non-swollen matrix boundary, while Equations (53) and (54) are the conditions at the gel/swelling agent interface. Volume increase over time is accounted for through a mass balance:

(55) $$\frac{\pi d^{2}}{4}\left(L-L_{0}\right)=\overset{L}{\underset{x^{*}}{\int }}\frac{\pi d^{2}}{4}\frac{M_{s}}{\rho }Sdx$$

where *d* is cylinder diameter, *L_0_* is the initial size of cylinder axis, *M_s_* is swelling agent molecular weight, and *ρ* is swelling agent density. The model is analytically solved by identifying suitable dimensionless variables and by assuming constant diffusion coefficients; results are in good agreement with experimental data concerning KCl release from HPMC tablets.

Korsmeyer et al. [124,125] proposed a model for the diffusion of a solute and a penetrant (water) within a swellable polymer slab, assuming non-constant diffusivities. Diffusion coefficients are expressed with a Fujita-type exponential dependence on penetrant concentration. At the beginning, the swelling occurs only along one dimension because of the glassy core of the devices; when a suitable plasticizer amount has reached the bulk, the sample relaxes isotropically and the swelling becomes three-dimensional. Changes in size are computed according the amount of absorbed solvent.

The mass balance for the penetrant can be written as follows adopting dimensionless variables:

(56) $$\frac{\partial C_{1}}{\partial \tau }=\frac{\partial }{\partial \xi }\left(D_{1}\frac{\partial C_{1}}{\partial \xi }\right)$$

(57) $$C_{1}=\frac{C_{w}}{C_{w,e}}$$

(58) $$\tau =\frac{tD_{1,s}}{L_{0}^{2}}$$

(59) $$\xi =\frac{x}{L_{0}}$$

where *C_1_* is the dimensionless water concentration, *D_1_* is water diffusion coefficient, *C_w_* is water concentration in the matrix, *C_w,e_* is water concentration in the matrix at equilibrium, *τ* is the dimensionless time, *D_1,s_* is water diffusion coefficient in the fully-swollen polymer, *L_0_* is the dry slab thickness, *ξ* is the dimensionless spatial coordinate, and *x* is the spatial coordinate. 

The mass balance for the solute is expressed as well in dimensionless terms:

(60) $$\frac{\partial C_{2}}{\partial \tau }=\frac{\partial }{\partial \xi }\left(D_{2}\frac{\partial C_{2}}{\partial \xi }\right)$$

(61) $$C_{2}=\frac{C_{s}}{C_{s,i}}$$

where *C_2_* is dimensionless drug concentration, *D_2_* is drug diffusion coefficient in the matrix, *C_s_* is drug concentration in the matrix, and *C_s,i_* is the initial drug concentration. The model can be solved by means of suitable initial and boundary conditions:

(62) $$C_{1}\left(0,\tau \right)=C_{1}\left(\xi^{\prime },\tau \right)=1$$

(63) $$C_{2}\left(0,\tau \right)=C_{2}\left(\xi^{\prime },\tau \right)=0$$

(64) $$C_{1}\left(\xi ,0\right)=0$$

(65) $$C_{2}\left(\xi ,0\right)=1$$

where 0 and ξ’ are the coordinates of slab surfaces. Diffusion coefficients are expressed as follows:

(66) $$D_{1}=D_{1,s}\exp\left[-\beta_{1}\left(1-C_{1}\right)\right]$$

(67) $$D_{2}=D_{2,s}\exp\left[-\beta_{2}\left(1-C_{1}\right)\right]$$

where *D_2,s_* is drug diffusivity in the fully-swollen matrix and *β_1_* and *β_2_* are constants. The complete model (with non-constant volume and diffusivities) was numerically solved and it showed a good agreement with experimental data in terms of theophylline release and dimensional changes of HPMC matrices. 

Ju et al. [126,127,128] developed a model that describes swelling and dissolution of HPMC matrix as well as drug release. The starting point is a mass balance in the following form, written in cylindrical coordinates:

(68) $$\frac{\partial \rho_{i}}{\partial t}=-\frac{\partial \rho_{i}}{\partial r}\frac{dr}{dt}+\frac{1}{r}\frac{\partial }{\partial r}\left(rD_{i}\rho_{t}\frac{\partial w_{i}}{\partial r}\right)-\frac{\rho_{i}}{V}\frac{dV}{dt}$$

where *ρ_i_*, *D_i_*, and *w_i_* are the mass concentration, the diffusion coefficient, and the mass fraction of the i-th component, respectively, *ρ_t_* is the overall mass concentration, *r* is the radial coordinate, *t* is time, and *V* is device volume. The first term on the right side accounts for the convection, which arises from the moving boundaries because of the swelling, the second term is related to the diffusion, and the third one takes into account the change in volume since the balance is written in terms of concentrations. Diffusion coefficients are expressed as a function of the concentration:

(69) $$\frac{D_{s,w}}{D_{w,0}}=k_{w}^{\prime }\exp\left(k_{w}w_{w}\right)$$

(70) $$\frac{D_{d}}{D_{d,0}}=\exp\left(-k_{d,p}w_{p}-k_{d,l}w_{l}-k_{d,d}w_{d}\right)$$

where *D_s,w_* is water self-diffusion coefficient, *D_d_* is drug mutual diffusion coefficient, *k_w_’* is a prefactor, *k_w_* and *k_d,i_* are weighting factors, and *D_i,0_* and *w_i_* are the diffusion coefficient at infinite dilution and the mass fraction of the *i*-th component, respectively (w, water; p, polymer; d, drug; l, lactose). Water diffusion coefficient *D_w_* is related to polymer volume fraction:

(71) $$D_{w}=D_{s,w}\upsilon_{2,s}.$$

**Figure 5:**
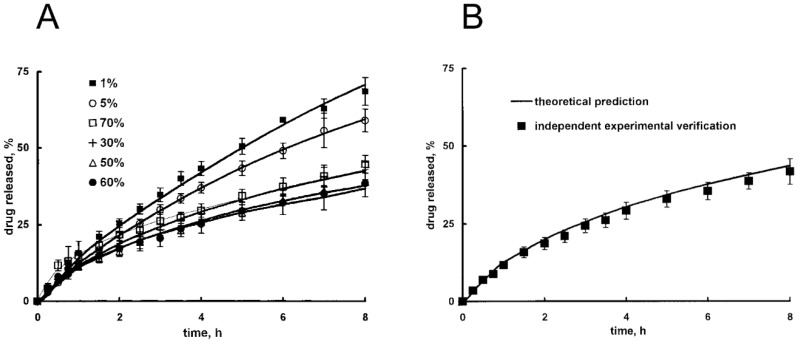
Release of theophylline as a function of time from hydroxypropyl methylcellulose (HPMC) matrices in phosphate buffer (pH 7.4) for different value of initial drug loading (expressed as w/w %) (**A**). Model validation through theoretical prediction of independent experimental data (theophylline release, initial drug loading 40%) (**B**). Adapted from Siepmann and Peppas [129], Copyright 2000 Springer Nature.

These authors also account for polymer dissolution. Siepmann and Peppas also developed a comprehensive model for HPMC devices that accounts for polymer swelling and dissolution. Mass balances are written in cylindrical coordinates, considering radial, axial, and azimuthal spatial coordinates; diffusion coefficients are expressed by means of free volume theory, with a Fujita-like exponential dependence on water content [129]. These authors tested model robustness by simulating drug release under various conditions, in terms of drug solubility, initial drug loading, and environmental pH (Figure 5A); the model was subsequently validated through a satisfactory comparison with independent experimental data (i.e., not employed for parameters fitting), as shown in Figure 5B. This verification confirms that a carefully tested model can be properly employed for device design, with in silico experiments that optimize experimental activity.

### 3.10. Chemically Controlled Systems

As mentioned (*vide supra*), when the system is in a chemically-controlled regime, the rate-determining phenomenon depends on system design. If the drug is covalently bound to polymer chains with a cleavable spacer, release mainly depends on the cleavage rate, because only unbound active molecules are free to diffuse. 

If bond scission occurs through hydrolysis, pseudo-first-order kinetics are a very good approximation due to the large water excess. Pitt and Schindler [130] developed a model for the release from a polymer film, assuming a constant diffusion coefficient and first order kinetics for bonds cleavage, and provided an analytical solution:

(72) $$\frac{\partial C\left(x,t\right)}{\partial t}=D\frac{\partial^{2}C\left(x,t\right)}{\partial x^{2}}+kC_{0}e^{-kt}$$

(73) $$C\left(x,t\right)=\frac{4kC_{0}}{\pi }\overset{\infty }{\underset{n=1}{\sum }}\frac{\left(e^{-\alpha_{n}t}-e^{-kt}\right)\sin\left[\frac{\left(2n-1\right)}{L}\pi x\right]}{\left(2n-1\right)\left(k-\alpha_{n}\right)}$$

(74) $$\alpha_{n}=\frac{{\left(2n-1\right)}^{2}\pi^{2}D}{L^{2}}$$

(75) $$\frac{Q_{t}}{Q_{\infty }}=\frac{8kD}{L^{2}}\overset{\infty }{\underset{n=1}{\sum }}\frac{\alpha_{n}^{-1}\left(1-e^{-\alpha_{n}t}\right)-k^{-1}\left(1-e^{-kt}\right)}{\left(k-\alpha_{n}\right)}$$

where *C* is drug concentration, *D* is diffusion coefficient, *k* is bond cleavage kinetic constant, *C_0_* is the initial concentration of bound drug, *x* is the spatial coordinate along to film thickness *L*, *Q_t_* and *Q_∞_* are the amount of drug released at time *t* and at infinite time, respectively. DuBose et al. [131] proposed and validated a statistical model that accounts for hydrogel swelling, degradation, and the release of a covalently-bound probe through hydrolytic cleavage; involved kinetics are assumed to be first order. Reid et al. [132] proposed models based on analytical theories and Monte Carlo simulations for the degradation of a gel based on Tetra-PEG polymers and the release of a covalently-bound drug. When bond scission is mediated by enzymes, a pseudo-first-order kinetic law can still a suitable choice for bond cleavage, as shown, for example, by Ehrbar and coworkers [133]. Notably, in order to describe properly experimental data, the authors needed to fit a different pseudo-first-order kinetic constant value for different polymer concentrations in the gel. This indicates that the obtained constants are affected by the diffusive limitations of the enzyme in the matrix and suggests that, in a more detailed modeling framework, enzyme diffusion should be explicitly considered. If more complicated kinetic laws are needed (such as Michaelis–Menten kinetics for enzyme), Equation (72) can be conveniently rewritten by substituting the second term on the right side with a suitable reaction rate. 

Bond cleavage can be performed also through photodegradation, as discussed by Griffin and coworkers [134] who synthesized PEG hydrogels where a drug mimetic compound (fluorescein) is covalently linked to the matrix with a photosensitive acrylated *ortho*-nitrobenzylether (*o*-NBE) moiety. This allows tailoring the release rate by tuning light intensity, duration of exposure, and wavelength. The time evolution of photodegradable groups *PD* as a function of time *t* is given by:

(76) $$PD=PD_{0}\exp\left(-\frac{\phi \varepsilon \lambda I\left(x,t\right)\left(2.303\cdot 10^{-6}\right)}{N_{A}hc}t\right)$$

where *PD_0_* is the concentration before light exposure, *ϕ* is quantum yield, *ε* is molar absorptivity, *I* is light intensity, *λ* is wavelength, *N_A_* is Avogadro number, *h* is Planck constant, and *c* is the speed of light. Lambert–Beer law is needed in order to account for light attenuation through the gel; this leads to a system of partial differential equations that can be numerically integrated (with suitable initial and boundary conditions) in order to determine drug-mimetic release.

**Figure 6:**
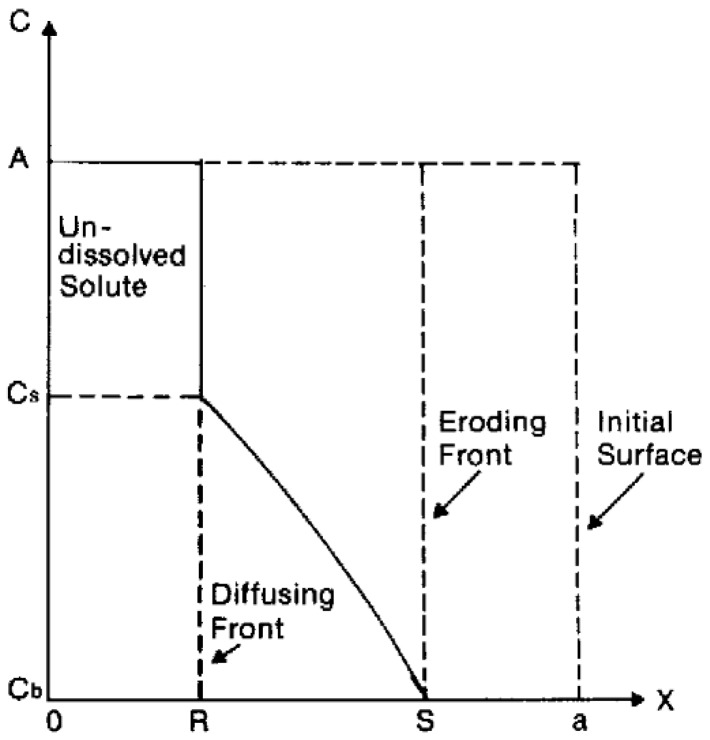
Schematization of a surface-eroding drug-eluting matrix through two moving fronts: An eroding front and a diffusing front. Reprinted from Lee [136], Copyright 1980 Elsevier.

A typical case of surface-eroding devices is constituted by hydrophobic polymer matrices (e.g., polyanhydrides) where water penetration rate is slower than water consumption due to chain scission; because of this, only the surface experiences degradation and erosion, while the bulk remains intact. Focusing on hydrogel, it is evident that this condition is not met because of the relevant water uptake; in this framework, surface erosion can be the rate-determining phenomenon when chain scission is mainly due to enzymes, which cannot appreciably diffuse into the matrix because of their high molecular weight and steric hindrance. In this regard, approaches developed for surface-eroding hydrophobic matrices can be also employed for enzymatically-degradable hydrogels [28,29,135]. Lee [136] introduced a mathematical model that identifies two moving fronts: An erosion front at matrix/water interface S (which moves at constant velocity) and a diffusion front R, which limits the thickness where drug concentration gradients are located. The model takes also into account the coexistence of dissolved and undissolved drug molecules. A model scheme is shown in Figure 6, where *C_s_* is drug solubility and *C_b_* is drug concentration in the surrounding medium.

This author also provided an analytical solution for a quantitative description of drug release:

(77) $$\frac{M_{t}}{M_{\infty }}=\delta +\frac{Ba}{D}\tau -\delta \frac{C_{s}}{A}\left(\frac{1}{2}+\frac{a_{3}}{6}\right)$$

(78) $$a_{3}=\frac{A}{C_{s}}+\delta h-\sqrt{{\left(\frac{A}{C_{s}}+\delta h\right)}^{2}-1-2\delta h}$$

(79) $$h=\frac{1}{2}\frac{Ba}{D}\left(1-\frac{A}{C_{s}}\right)$$

(80) $$\delta =\frac{S-R}{a}$$

(81) $$\tau =\frac{Dt}{a^{2}}$$

(82) S(t)=a−Bt

where *M_t_* and *M_∞_* are the amount of drug released at time *t* and at infinite time, respectively, *A* is the initial drug concentration (assumed to be above solubilization limit), *B* is the surface erosion kinetic constant (expressed as a velocity), *δ* is the relative separation between erosion and diffusion fronts, and *τ* is the dimensionless time. In particular, the parameter *Ba*/*D* indicates the relative contribution of erosion and diffusion to drug release. The model prediction is very close to zero-order release kinetics when the initial drug amount is much higher than solubilization limit.

Hopfenberg [137] proposed a semi empirical model for drug release from surface-eroding slabs, cylinders, and spheres:

(83) $$\frac{M_{t}}{M_{\infty }}=1-{\left(1-\frac{k_{a}t}{C_{0}a_{0}}\right)}^{n}$$

where *k_a_* is the erosion rate constant, *C_0_* is initial drug concentration, *a_0_* is initial characteristic dimension (half-thickness for slabs, radius for cylinders and spheres), and *n* is an exponent that accounts for system geometry, equal to one, two, and three for slabs, cylinders, and spheres, respectively. Katzhendler et al. [138] further extended the model considering two-dimensional erosion, along radial and axial coordinates:

(84) $$\frac{M_{t}}{M_{\infty }}=1-{\left(1-\frac{k_{a}t}{C_{0}a_{0}}\right)}^{2}\left(1-\frac{2k_{b}t}{C_{0}b_{0}}\right)$$

where *k_a_* and *k_b_* are the radial and axial erosion kinetic constants, respectively, and *a_0_* and *b_0_* are the initial radius and thickness of the tablet, respectively. Since degradation can be also modeled as a stochastic process, Göpferich and Langer [139] adopted Monte Carlo simulations to describe surface-eroding devices. A two-dimensional grid is divided in pixels that assume a different numerical value according to their possible states: Crystalline polymer, amorphous polymer, eroded polymer. Pixel erosion is a random process that obeys a first-order Erlang probability density function.

Focusing on bulk-degrading system, the mathematical model must include a suitable description of chain scission kinetics as well as its impact on drug diffusivity, which dynamically increases. 

Mason et al. [140] discussed a model aimed at describing the degradation of hydrogels made of polylactic acid-polyethylene glycol-polylactic acid (PLA-PEG-PLA) triblock macromers; chain scission is due to hydrolysis of ester bonds that constitute PLA backbone. Degradation kinetics are assumed to be of first order with respect to the polymer, according to the following mass balance:

(85) $$\frac{dn_{E}}{dt}=-k_{E}^{\prime }n_{E}$$

where *n_E_* is the number of moles of ester bonds and *k_E_’* is the pseudo first order kinetic constant for the hydrolysis of ester bonds. The authors adopted scaling laws for highly swollen gels (*ν_2,s_* < 0.1) to relate structural changes and diffusion increase to polymer degradation:

(86) $$M_{c}\sim e^{2jk_{E}^{\prime }t}$$

(87) $$Q\sim e^{\frac{6}{5}jk_{E}^{\prime }t}$$

(88) $$\xi \sim e^{\frac{7}{5}jk_{E}^{\prime }t}$$

(89) $$1-\frac{D_{g}}{D_{0}}=\frac{r_{s}}{\xi }\sim e^{-\frac{7}{5}jk_{E}^{\prime }t}$$

where *j* is the number of ester bonds per PLA block. The model has been successfully validated through comparison with experimental data, in terms of volumetric swelling ratio and bovine serum albumin (BSA) release.

In general, different modeling frameworks can be formulated according to the underlying cross-linking mechanisms, namely chain-growth or step-growth polymerization [141]. In the chain-growth process, polymerization starts from reactive centers (like radicals) that propagate. This leads to covalently cross-linked high molecular weight kinetic chains, but also to low conversion of reactive functional groups and network non-idealities that negatively affect mechanical properties and drug release. Step-growth gelation is characterized by the presence of at least two different multifunctional monomers (i.e., average monomer functionality higher than 2) with mutually reactive functional groups. This process allows a better cross-linking control, leading on the one side to improved material performances and, on the other side, to more accurate modeling predictions. The influence of cross-linking process on the properties of PEG-based hydrogels has been discussed by Tibbitt and coworkers [142]. 

A statistical model for degradation of hydrogels from chain-growth cross-linking was proposed by Metters and coworkers [143] for PLA-PEG-PLA networks. Assuming that PLA units are hydrolyzed according to a pseudo-first order kinetics (Equation (91)), the probability that a random PLA unit has been hydrolyzed *P* can be obtained by:

(90) $$PLA=PLA_{0}\exp\left(-kt\right)$$

(91) $$P=1-\frac{PLA}{PLA_{0}}=1-\exp\left(-kt\right)$$

where *PLA_0_* is the amount of PLA units before degradation onset, *k* is pseudo-first-order hydrolysis constant, and *t* is time. In this system, each PEG chain is attached to the gel by two PLA units; each PLA–PEG–PLA segment can thus exist in three states: (1) Completely attached (PLA units intact), (2) one unit attached and one hydrolyzed (dangling segment), and (3) both units hydrolyzed (segment released from the network). The fractions of each group can be computed as follows:

(92) $$y_{1}={\left(1-P\right)}^{2}$$

(93) $$y_{2}=2P\left(1-P\right)$$

(94) $$y_{3}=P^{2}$$

where *y_i_* is the fraction of the segments in the *i*-th state. From Equations (92)–(94) it is possible to estimate the probability that a chain connected to N cross-links can be released, and thus the mass loss as a function time. An improved version of the model, which accounts for network non-idealities, was subsequently proposed by the authors [144].

For what is regarded as step growth cross-linking, a statistical model was proposed by Metters and Hubbel [145] and applied to different systems, such as gels made of PEG-tetra-norbornene macromer (PEG4NB) (a tetrafunctional monomer) and dithiothreitol (DTT) (a bifunctional cross-linker), whose photopolymerization leads to a network with thiol-ene cross-links and hydrolysable ester bonds [146]. Focusing on this system, and assuming a pseudo-first-order kinetics for ester bonds hydrolysis, the fraction of hydrolyzed ester groups *P_ester_* as a function of time *t* is given by:

(95) $$P_{ester}=1-\exp\left(-kt\right)$$

where *k* is the pseudo-first order kinetic constant. The fraction of intact elastic chains is given by:

(96) $$1-P_{chain}={\left(1-P_{ester}\right)}^{N}=\exp\left(-Nkt\right)$$

where *N* is the number of degradable units (in this specific example, ester bonds) connected to one elastic chain (equal to 2 for this system). Assuming an ideal step-growth network, the fraction of PEG4NB macromer with *i* arms connected to the network as a function of time *F_i,fA_* can be computed as follows:

(97) $$F_{i,f_{A}}=\frac{f_{A}!}{\left(f_{A}-i\right)!i!}P_{chain}^{\left(f_{A}-i\right)}{\left(1-P_{chain}\right)}^{i}$$

where *f_A_* is the number of macromer reactive functionalities (equal to 4 for PEG4NB). Cross-linking density as a function of time during degradation can be thus obtained:

(98) $$\nu_{c}=\left(\overset{i=3}{\underset{f_{A}}{\sum }}\frac{i}{2}F_{i,f_{A}}\right)A_{0}$$

where *A_0_* is macromer concentration at swelling equilibrium before degradation onset; in the summation, *i ≥ 3* since macromers with only two arms connected with elastic chains form an extended loop rather than a cross-link. Although specific examples are here reported, both degradation models can be extended to different systems, in terms of composition and degradation mechanisms. Their application to drug delivery is immediate once the diffusion coefficient takes into account the dynamic degradation.

It is worth mentioning that chain scission can also take place through photodegradation. Tibbitt et al. [147] synthesized polymer networks with photodegradable cross-links and proposed a kinetic model for photocleavage reactions:

(99) $$\frac{\partial I\left(z,t\right)}{\partial z}=-2.3I\left(z,t\right)\underset{i}{\sum }\varepsilon_{i}C_{i}\left(z,t\right)$$

(100) $$\frac{\partial C_{i}\left(z,t\right)}{\partial t}=-\frac{\varphi \varepsilon_{i}\lambda }{N_{A}hc}C_{i}\left(z,t\right)I\left(z,t\right)$$

(101) $$\frac{\partial C_{j}\left(z,t\right)}{\partial t}=\frac{\varphi \varepsilon_{i}\lambda }{N_{A}hc}C_{i}\left(z,t\right)I\left(z,t\right)$$

where *z* and *t* are spatial coordinate and time, respectively, *I* is local irradiation intensity, *ε_i_* is molar absorptivity of the *i*-th photoactive species, *Ci* is the concentration of the *i*-th photoactive species, *φ* is quantum yield, *λ* is wavelength, *N_A_* is Avogadro number, *h* is Planck constant, *c* is speed of light, and *Cj* is the concentration of the *j*-th cleavage product. Equation (90) is Lambert–Beer law, which accounts for light intensity attenuation through the gel; degradation kinetics are assumed to be of the first order. Equations (99)–(101) can be numerically solved with suitable initial and boundary conditions; they were coupled with a statistical model for the description of network structure, based on the modeling framework of Metters and coworkers for chain-growth cross-linking [143]. The overall model was validated through comparison with experimental data; although the authors did not consider drug release, the diffusion coefficient can be correlated to network degradation with a suitable expression, as mentioned.

Monte Carlo simulations can be also adopted for this kind of systems; Siepmann and coworkers [148] proposed a model for bulk-eroding polylactic-*co*-glycolic acid (PLGA) particles, similar to the one introduced by Göpferich and Langer. The microsphere is divided in pixels, which can assume two values: 0 for pores and 1 for non-eroded polymer. Drug diffusion is computed through a mass balance, where diffusion coefficient values are expressed as a function of a time and position dependent porosity, which depends on the number of degraded pixels. Vlug-Vensink et al. [149] developed and validated a model based on Monte Carlo simulations for the release of proteins and liposomes from cross-linked dextran particles. 

Hydrogels can also be designed so that loaded active molecules can be non-covalently bound to polymer chains, through electrostatic interactions or hydrophobic effects. In this case, the binding must be accounted for in the model in order to assess its impact on the release rate. Binding can be taken into account in the expression for diffusion coefficients or by explicitly including adsorption and desorption rates in the mass balances.

Focusing on the first approach, Fatin-Rouge et al. [27] developed a model for the evaluation of the diffusion coefficients of small ions in agarose gel, including steric, chemical, and electrostatic interactions. Diffusivity values account for the formation of non-covalent complexes between the diffusant and gel sites. The model of Fatin-Rouge and coworkers takes into account, in detail, several contributions but it was conceived for small ions, which limits its applicability in the drug delivery field when more complicated compounds are used. To authors’ best knowledge, analogous models for macromolecules are not available in literature. This may be due to the attainment of complex interactions between the solute and the matrix (electrostatics, Van der Waals, π–π interactions, etc.), which are challenging to assess and to include formulaically.

Peppas and Wright [150] investigated the diffusion of theophylline, vitamin B_12_, and myoglobin in interpenetrating PVA/PAA networks at different pH values. Experimental data showed a further reduction of diffusivity values due to the binding, corroborated by the change of attenuated total reflectance-Fourier transform infrared (ATR-FTIR) spectroscopy. The authors proposed a modified version of Peppas and Reinhardt theory (Equation (32)):

(102) $$\frac{D_{g}}{D_{0}}=k_{1}\left(\frac{M_{c}-M_{c}^{*}}{M_{n}-M_{c}^{*}}\right)\exp\left(-\frac{k_{2}r_{s}^{2}}{Q-1}\right)-k_{3}\exp\left(\frac{\Delta H^{AB}}{kT}\right)$$

where *k_3_* is a constant that depends on both polymer and solute and Δ*H^AB^* is a term proportional to the shift in spectral frequency of FTIR spectra.

Liu et al. [151] studied the diffusion of water-soluble drugs in gels made of 2-hydroxyethylmethacrylate/methacrylic acid; experimental data evidenced the presence of solute/matrix interactions. The authors developed an expression for diffusion coefficients that accounts for the binding:

(103) $$\frac{D_{i}}{D_{i0}}=\frac{F_{i}S_{i}}{1+\sum_{j}K_{ij}\frac{\varphi_{2j}}{\varphi_{1}}}$$

where *D_i_* and *D_i0_* are the diffusion coefficients of the drug *i* in the gel and in water solution, respectively, *Fi* are *Si* are hydrodynamic and steric resistance factors (computed using large-pore effective medium theory), *K_ij_* is Henry adsorption constant of drug *i* relative to polymer component *j*, *φ_2j_* is the volume fraction of polymer component j, and *φ_1_* is water volume fraction.

As mentioned, solute binding can be also considered in the mass balance itself, through a diffusion/reaction approach. In this case, diffusion coefficient can be estimated from suitable correlations, while adsorption and desorption rate constants, or the equilibrium constant, can be obtained from experimental data fitting. Assuming a reversible binding, and that reaction is faster than diffusion, the following mass conservation equation can be employed [29]:

(104) $$\frac{\partial C_{p}}{\partial t}=\frac{D}{K_{b}+1}\nabla^{2}C_{p}$$

(105) $$K_{b}=\frac{C_{pl}}{C_{p}}$$

where *C_p_* and *C_pl_* are the concentration of free and bound solute, respectively, *D* is the diffusion coefficient, and *K_b_* is the equilibrium constant for the binding, which is assumed to be time-independent. According to the adopted hypothesis (reversible binding, rapid binding equilibrium, *K_b_* independent on time), Equation (104) states that binding slows down diffusion by a factor equal to *K_b_ + 1*. However, this model can be applied only to simple systems because of the underlying assumptions.

Singh et al. [152] developed a model that combined diffusion and desorption by means of Langmuir adsorption isotherm. The authors performed parametric simulations in order to understand the impact of affinity constant, desorption rate, and the maximum amount of drug that can be bound to the matrix. A similar approach was adopted by Rossi and coworkers [153], although they determined experimentally the parameters for the Langmuir adsorption isotherm. The authors also proposed an expression for the diffusion coefficient that accounts for adsorption:

(106) $$\frac{D}{D_{0}}=\frac{\varepsilon }{\varepsilon +\left(1-\varepsilon \right)\frac{q^{\infty }K}{{\left(1+KC_{G}\right)}^{2}}}$$

where *D* and *D_0_* are diffusion coefficient in the gel and in water solution, respectively, *ε* is gel porosity, *q^∞^* is the maximum adsorbed concentration, *K* is equilibrium constant, and *C_G_* is diffusant concentration in the gel.

Vulic et al. [154] focused their attention on the release of recombinant human fibroblast growth factor 2 (rfFGF2) and chondroitinase ABC (ChABC) from hydrogels made of hyaluronan and methyl cellulose functionalized with protein-binding peptides. They described drug release through a mathematical model that explicitly includes adsorption and desorption rates. In particular, they highlighted three dimensionless parameters (*α*, *β,* and *γ*) that mainly determines system behavior in terms of release rate:

(107) $$\alpha =\frac{C_{pro,0}}{C_{pep,T}}$$

(108) $$\beta =\frac{L^{2}k_{off}}{D}$$

(109) $$\gamma =\frac{C_{pro,0}}{K_{D}}$$

where *C_pro,0_* is the amount of free (unbound) protein before release onset, *C_pep,T_* is the total amount of peptides, *L* is gel characteristic length, *D* is protein diffusion coefficient, *k_off_* is desorption rate constant, and *K_D_* is affinity equilibrium constant. The parameter *α* is representative of the free protein in the gel, *β* is the ratio between diffusion and desorption characteristic time scales, and *γ* is related to the amount of bound protein before release onset. The investigated system is characterized by β >> 1 (diffusion-controlled system) and γ << 1 (small fraction of bound protein). The model was validated with experimental data and the authors performed parametric simulations by varying the affinity equilibrium constant and adsorption/desorption rate constants. Such analysis is useful to evaluate how the release rate is affected by protein/matrix interactions and provide useful guidelines for the design of new devices, where affinity can be tuned in order to achieve the desired release profile.

Sakiyama-Elbert and Hubbel [155] developed an affinity hydrogel by copolymerizing fibrin with heparin-binding peptides, loaded with heparin and basic fibroblast growth factor (bFGF), a heparin-binding growth factor. Into the matrix, heparin can exist in three different states: Bound to the matrix (by means of electrostatic interactions that occur with heparin-binding peptides), bound to basic fibroblast growth factor, or as a free molecule. The growth factor can diffuse freely as well or it can be adsorbed on the matrix through the electrostatically bound heparin. This led to the following kinetic scheme, assuming reversible binding:

(110) G+H↔GH

(111) G+HP↔GHP

(112) H+P↔HP

(113) GH+P↔GHP

where *G* is free growth factor, *H* is free heparin, *P* is heparin-binding peptide, *GH* is growth factor/heparin complex, *HP* is heparin/peptide complex, and *GHP* is growth factor/heparin/peptide complex. Assuming that *k_f_* and *k_r_* are association and dissociation rate constants for bFGF binding to heparin (Equations (110) and (111), respectively), and *κ_f_* and *κ_r_* are the analogous constants related to peptide binding to heparin (Equations (112) and (113), respectively), a mass balance for each component can be written:

(114) $$\frac{\partial C_{G}}{\partial t}=D_{G}\frac{\partial^{2}C_{G}}{\partial x^{2}}-k_{f}C_{G}C_{H}+k_{r}C_{GH}-k_{f}C_{G}C_{HP}+k_{R}C_{GHP}$$

(115) $$\frac{\partial C_{H}}{\partial t}=D_{H}\frac{\partial^{2}C_{H}}{\partial x^{2}}-k_{f}C_{G}C_{H}+k_{r}C_{GH}-\kappa_{f}C_{H}C_{P}+\kappa_{R}C_{HP}$$

(116) $$\frac{\partial C_{P}}{\partial t}=-\kappa_{f}C_{H}C_{P}+\kappa_{r}C_{HP}-\kappa_{f}C_{GH}C_{P}+\kappa_{R}C_{GHP}$$

(117) $$\frac{\partial C_{GH}}{\partial t}=D_{GH}\frac{\partial^{2}C_{GH}}{\partial x^{2}}+k_{f}C_{G}C_{H}-k_{r}C_{GH}-\kappa_{f}C_{GH}C_{P}+\kappa_{R}C_{GHP}$$

(118) $$\frac{\partial C_{HP}}{\partial t}=\kappa_{f}C_{H}C_{P}+\kappa_{r}C_{HP}-k_{f}C_{G}C_{HP}+k_{R}C_{GHP}$$

(119) $$\frac{\partial C_{GHP}}{\partial t}=k_{f}C_{G}C_{HP}+k_{r}C_{GHP}-\kappa_{f}C_{GH}C_{P}+\kappa_{R}C_{GHP}$$

where *Ci* is the concentration of the *i*-th component and *D_i_* is the diffusion coefficient of the *i*-th component; it is here assumed that the compounds bound to the matrix cannot diffuse. The model constituted by Equations (114)–(119) can be solved with suitable initial and boundary conditions and parameters values (in terms of kinetic constants and diffusion coefficients). Model results qualitatively agree with experimental data, although a systematic comparison was not performed. 

Yan and Casalini [156] investigated the release of bone morphogenetic protein 2 (BMP2), a bone growth factor, from hydrogels made of hyaluronic acid (HA); in particular, experimental data showed that the eluting rate depends on the initial microenvironmental pH inside the gel (neutralization occurs during the release, which is performed in buffer solution). Indeed, while the release at pH 7 could be well-described by means of a simple diffusion equation (Fick’s second law), it was necessary to assume the attainment of solute/matrix interactions to account for the release rate when the initial pH inside the gel is 4.5. This hypothesis was verified and corroborated by means of molecular dynamics simulations, using a model system consisting of a single BMP2 protein interacting with a short HA chain. Molecular modeling showed that at pH 4.5, BMP2 is strongly bound to the matrix by means of electrostatic interactions, due to the protonation state of the protein, whose overall charge is positive while HA is still largely dissociated (and thus negatively charged). At neutral pH, BMP2 overall charge is negative and exhibits weak Van der Waals interactions and electrostatic repulsion with the negatively-charged matrix. System behavior was thus rationalized as follows: When the initial pH is 4.5, most of the protein is adsorbed on polymer chains before release onset, because of electrostatic interactions. When release and gel neutralization begin, the bound protein is irreversibly desorbed by virtue of electrostatic repulsion, due to the change in its protonation state; therefore, BMP2 release rate is due to the synergy between desorption and diffusion, whose characteristic time scales are comparable. Mass balances can be formulated as follows:

(120) $$\frac{\partial C_{protein,u}}{\partial t}=D\frac{\partial^{2}C_{protein,u}}{\partial z^{2}}+k_{des}C_{protein,b}-k_{ads}C_{protein,u}$$

(121) $$\frac{\partial C_{protein,b}}{\partial t}=-k_{des}C_{protein,b}+k_{ads}C_{protein,u}$$

where *C_protein,u_* and *C_protein,b_* are the concentrations of unbound and bound protein, respectively, *D* is the diffusion coefficient, and *k_ads_* and *k_des_* are adsorption and desorption kinetic constants, respectively. Model fitting showed that only desorption plays a key role, since adsorption constant is eight orders of magnitude less than desorption one. At pH 7, essentially the entire protein is unbound and free to move in the water-filled pores: In this case, only molecular diffusion matters and Fick’s second law provides a good agreement with data. 

## 4. Conclusions

About 60 years have passed since the publication of the seminal work of Wichterle and Lim: The interest concerning the applications of hydrogels in biomedical field did not fade out, and neither did the development of new and exhaustive modeling approaches. The use of mass conservation equations, one of the fundamental pillars in chemical engineering, is still the method of choice for determining the main release mechanism as well as release kinetics, which are essential to evaluate the performances of the pharmaceutical formulation of interest. Methods at molecular scale opened new possibilities to understand the impact of available degrees of freedom (cross-link density, polymer composition, ionization degree, etc.) on network structure and transport phenomena, thanks to their intrinsic capability to account for the interplay between hydrophilic/hydrophobic effects, electrostatic interactions, and environmental effects (salt concentration, pH, etc.). While such effects were lumped in some parameters included in macroscale models, validated molecular dynamics simulations and coarse-grained models can provide a trend-wise or even a quantitative evaluation of the effect of design parameters, including information that are challenging or impossible to obtain experimentally. 

Their growing application in the drug delivery field cannot be underestimated, since they allow an in silico device design and optimization that starts at fundamental molecular level, with an unprecedented level of detail, and culminates with a device with tailored properties and the desired release rate. The relevance of this approach has been recently internationally acknowledged within the “safety by design” paradigm, meant to become the reference standard when designing new controlled drug delivery systems.

## Figures and Tables

**Table 1:** Summary of modeling approaches and their main applications.

Method	Time Scale	Length Scale	Applications
Molecular dynamics simulations	Nanoseconds	Nanometers (tenth)	Transport phenomena Chain conformation
Coarse-grained models	Microseconds	Nanometers (up to hundredth)	Transport phenomena Network structure Swelling equilibrium
Macroscale models	Seconds	Meters	Structural parameters Swelling dynamics Degradation kinetics Release rate

**Table 2:** Values of exponent n for different geometries. Taken from Siepmann and Peppas [107].

n [-]			Release Mechanism
Thin Film	Cylinder	Sphere	
0.5	0.45	0.43	Fickian diffusion
0.5 < *n* < 1.0	0.45 < *n* < 0.89	0.43 < *n* < 0.85	Anomalous transport
1.0	0.89	0.85	Case II transport

**Table 3:** Models for diffusion coefficients in hydrogels.

Underlying Theory	Expression	Reference
Free volume	$\frac{D_{g}}{D_{0}}=k_{1}\left(\frac{M_{c}-M_{c}^{*}}{M_{n}-M_{c}^{*}}\right)\exp\left(-\frac{k_{2}r_{s}^{2}}{Q-1}\right)$	[112]
Free volume	$\frac{D_{g}}{D_{0}}=\left(1-\frac{r_{s}}{\xi }\right)\exp\left[-Y\left(\frac{\upsilon_{2,s}}{1-\upsilon_{2,s}}\right)\right]$	[113]
Hydrodynamic	$\frac{D_{g}}{D_{0}}=\exp\left[-\left(\frac{3\pi L_{c}N_{A}}{M_{n}\ln\left(\frac{L_{c}}{2r_{f}}\right)}\right)r_{s}\upsilon_{2,s}^{1/2}\right]$	[114]
Hydrodynamic	$\frac{D_{g}}{D_{0}}={\left[1+{\left(\frac{r_{s}^{2}}{k}\right)}^{1/2}+\frac{1}{3}\frac{r_{s}^{2}}{k}\right]}^{-1}$	[115]
Obstruction	$\frac{D_{g}}{D_{0}}=\exp\left[-\frac{r_{s}+r_{f}}{r_{f}}\upsilon_{2,s}^{1/2}\right]$	[117]
Obstruction	$\frac{D_{g}}{D_{0}}=e^{-\alpha }+\alpha^{2}e^{\alpha }E_{1}\left(2\alpha \right)$	[118]
Obstruction	$\frac{D_{g}}{D_{0}}={\left(1+\frac{2}{3}\alpha \right)}^{-1}$	[119]
Obstruction	$\frac{D_{g}}{D_{0}}=\exp\left[-\pi {\left(\frac{r_{s}+r_{f}}{k_{s}\upsilon_{2,s}^{1/2}+r_{f}}\right)}^{2}\right]$	[120]
Obstruction/hydrodynamic	$\frac{D_{g}}{D_{0}}=\frac{\exp\left[-0.84\alpha^{1.09}\right]}{\left[1+{\left(\frac{r_{s}^{2}}{k}\right)}^{1/2}+\frac{1}{3}\frac{r_{s}^{2}}{k}\right]}$	[121]
Obstruction/hydrodynamic	$\frac{D_{g}}{D_{0}}={\left(1+\frac{2}{3}\alpha \right)}^{-1}\exp\left[-\pi \upsilon_{2,s}^{0.174ln\left(59.6\frac{r_{f}}{r_{s}}\right)}\right]$	[122]
